# Supplementary material for: Market-oriented job skill valuation with cooperative composition neural network
Source: Nat Commun. 2021 Mar 31;12:1992. doi: 10.1038/s41467-021-22215-y (PMC8012576; doi:10.1038/s41467-021-22215-y)
Supplement: Supplementary file 1 — Supplementary Information [file 41467_2021_22215_MOESM1_ESM.pdf]

Market-oriented Job Skill Valuation with Cooperative Composition Neural Network

Supplementary Information

Supplementary Note 1: Related Works

Supplementary Explanations: Terms and Notations (Table S1 - S2)

Supplementary Note 2: Data Preprocessing

Supplementary Note 3: Level Influence Bias Estimation

Supplementary Experiment 1: Data Analysis (Table S5 - S6, Figure S1 - S4)

Supplementary Experiment 2: Parameter Experiments (Table S7, Figure S5)

Supplementary Experiment 3: Experiments on the Designer Dataset (Table S8, Figure S6 - S7)

Supplementary Experimental Results (Figure S8 - S9, Table S9)

Supplementary Tables: Numerical Statistics of the Original Figures (Table S10 - Table S23)

Supplementary References

Supplementary Note 1: Related Works

Data-driven Labour Market Analysis

Labour market analysis can help the government to understand the demand and supply of labour market, which is essential for making timely economic decisions. For a long time, surveys have been collected for labour market analysis[1–5]. However, due to the dynamic and indistinct nature of labour market, it is usually difficult and cost-intensive for surveys to support fine-grained and up-to-date analysis. The idea of using job advertisements for labour market analysis can be traced back to the 1990s, when job postings collected from newspapers have been studied to reveal market demand[6]. In the era of the Internet, online recruitment services have been accumulating abundant job advertisement data[7,8], which provides an unparalleled chance for catching the labour market dynamics in an automatic and cost-efficient manner. Based on these online services, researchers on Labour Market Intelligence[9] have made remarkable efforts on collecting and processing vast online job vacancies for timely labour market analysis[10–13]. Along this line, in recent years, a variety of machine learning techniques have been adopted to further discover knowledge from the job advertisements[14]. For example, boselli *et al.*[9,12] adopted text classification techniques to automatically classify job advertisements into standard occupations for occupation analysis. Wu *et al.*[15] adopted Tensor Factorization for job demand prediction. Marrara *et al.*[16] proposed a language model-based approach to discover new occupations in the market. Besides, latent factor models have also been widely adopted to discover market demand patterns[17,18]. Different from existing methods, in this work, we proposed a machine-learning-based method to measure the value of job skills by modeling their relations with job salary.

Data-driven Salary Prediction

For a long time, researchers have been studying factors that affect job salary[5,19–21]. However, although various factors have been found to be correlated with salary, quantitative salary prediction is still a challenging task. In recent years, machine learning technology has been adopted for data-driven salary prediction, which aims to predict job salary in a quantitative and explicit way. For example, Lazar *et al.*[22] adopted Support Vector Machine (SVM) on survey data to predict job salary. Blankmeyer *et al.*[23] proposed a Bayesian model to model peer-group interaction for salary benchmarking on the nursing facility. Lin *et al.*[24] proposed a topic model to jointly model job salary and online public opinions. Although these works have developed effective models for specific scenarios, few of them fit for the application scenario of this paper, which predicts the salary for job openings in the job market. To the best of our knowledge, the most state-of-the-art salary prediction model on the job posting data is proposed by Meng *et al.*[25], which developed a Holistic Salary Benchmarking Matrix Factorization (HSBMF) model for predicting the missing salary information. Therefore, we regard HSBMF as a baseline method of our model. However, since HSBMF is a salary benchmarking method that cannot handle the complicated skill requirements and job contexts, it cannot perform well in our task. To better validate our model, similar to Lazar *et al.*[22], we also adopted classical machine learning algorithms (i.e., SVM, Linear Regression, GBDT) as comparisons, which are widely-adopted in various real-world prediction tasks.

Attentive Neural Networks

Attentive neural networks were first applied in the field of Computational Vision (CV), where the models simulate human attention mechanisms to distinguish the important parts of the input image[26,27]. Then, in the field of Computational Linguistics, attention mechanism is applied on text data, which strongly boosted the performances of various tasks, such as machine translation[28,29], text classification[30], and question answering[31]. Later, the success of attentive networks spreads rapidly to the field of temporal sequential modeling[32,33]. In these tasks, attention mechanism is adopted to distinguish time periods that have a strong influence on the future trend of the sequences. In addition to these regular data formats, the attention mechanism is also applied to graph data[34–37]. Specifically, the graph attentive networks can recognize the importance of neighbors when extracting the local representation of a node. Recently, owing to the high flexibility and interpretability of attention mechanism, attentive neural networks have been widely applied in various domains of real-world applications, such as disease risk prediction[38], traffic estimation[39], knowledge tracing[40], and fraud detection[41]. In this work, we adopted the attention mechanism to distinguish the importance of different skills required by a job posting. This not only raises the model's predicting performance but also supports explainable skill value assessment.

Supplementary Explanations: Terms and Notations

Supplementary Table S1. Concept Explanation. This table explains the meaning of the concepts and terms appearing in this paper. Although some of these concepts lack formal definitions, they frequently appear in related literature.

Concept	Explanation
Salary system	A system that employers use to determine an employee's compensation[42,43].
Technological changes	The overall process of invention, innovation, and diffusion of technology[18,44].
Skills gap	The disparity between the quality and adequacy of skills possessed by labours in the market and required by the employer[7,8,45].
Talent	In this work, talent represents skilled labours who own advanced job skills or work in high-skilled occupations. This term is commonly used in recent related works[46–48].
Job skill	In this work, we use the term job skill to denote the ability to do a specific kind of task in different jobs. Following previous works[15,49,50], we use skill words in the job postings as observations of skill requirements.

Supplementary Table S2. Notations in this paper. This table lists the major notations of this paper and explain their meanings.

Notation	Explanation
$\mathcal{J}$	A set of job postings.
J_j	The $j\text{-}th$ job posting.
$\mathbf{C_j}$	The contextual information of the $j\text{-}th$ job posting.
$\mathbf{S_j}$	The skill requirement of the $j\text{-}th$ job posting.
$\mathbf{Y_j}$	The salary range of the $j\text{-}th$ job posting.
y_j^l	The lower-bound salary of the $j\text{-}th$ job posting.
y_j^u	The upper-bound salary of the $j\text{-}th$ job posting.
$s_j^{(i)}$	The $i\text{-}th$ required skill in the $j\text{-}th$ job posting.
$\mathrm{lv}_j^{(i)}$	The level that describes the $i\text{-}th$ required skill in the $j\text{-}th$ job posting.
$\mathbf{A}$	The skill graph.
T	The number of time intervals.
$\mathbf{W}^*$	Model parameters.
$\mathbf{b}^*$	Model parameters.
$(\mathbf{E^s})^{(\mathbf{t})}$	The skill embedding vectors of the $t\text{-}th$ time interval.
$\mathbf{e^s}$	The embedding vector of skill s.
$\mathbf{o_i^c}$	The $i\text{-}th$ continuous context input vector.
$\mathbf{o_i^d}$	The one-hot representation of the $i\text{-}th$ discrete context input.
$\mathbf{e_i^c}$	The projected feature vector of the $i\text{-}th$ continuous context.
$\mathbf{e_i^d}$	The embedding vector of the $i\text{-}th$ discrete input.
$\mathbf{h_1}$	The first-order interaction extracted by the linear projection.
$\mathbf{h_2}$	The second-order interaction extracted by the multiplicative operation.
$\mathbf{x^{(k)}}$	The output of the $k\text{-}th$ MLP layer.
v^l	The lower-bound skill value.
v^u	The upper-bound skill value.
p	The gap between the lower-bound value and upper-bound value.
$\mathbf{U^{(k)}}$	The input of all the nodes in the $k\text{-}th$ GCN layer.
a_i	The domination of the $i\text{-}th$ skill.
$\tilde{y}_j^l$	The estimated lower-bound salary of the $j\text{-}th$ job.
$\tilde{y}_j^u$	The estimated upper-bound salary of the $j\text{-}th$ job.

Supplementary Note 2: Data Preprocessing

Data Source

In this work, we utilized two datasets to validate our model, which consist of IT-related job postings and designer-related job postings, respectively. The job postings were collected from an online recruitment website, namely Lagou (https://www.lagou.com/), where recruiters can post their job openings to attract candidates. In this website, each job opening is displayed in HTML, which contains structured information of salary range, company, location, time, and job description text. We parsed the HTML and obtained structured job posting information. In addition to the job postings, we also collected a company dataset from the same website, which contains information of the recruiting companies. We used this dataset to build company features for the model. Table S3 shows some examples of the company records.

Skill and Level Extraction

To extract the skill words, we first applied word segmentation to the job descriptions with *Jieba*[51]. Next, we combined the words into n-grams ($n \leq 4$) and filtered out the n-grams that contain stopwords or have low appearing frequencies. Then, we manually dropped the n-grams that represent no skills and merged the rest according to their meanings. Finally, we regarded the rest n-grams as skill words and built a skill dictionary with them. To extract the level words, we first counted the frequency of each n-gram appearing in front of a skill word. Then, we ranked these n-grams according to the frequency and regarded the top n-grams as candidates. Finally, we manually selected the n-grams that represent levels and built a level dictionary with them.

Duplication Processing

We regarded two job postings as duplicated if they meet all the following conditions:

- They are posted at the same time period. Specifically, we regard each half a year as a time period in our experiment.
- They have the same contextual information (i.e., working address, title, experience, etc.) and salary range.
- They have highly-similar job descriptions. Specifically, we first utilized text embedding to roughly discover similar job descriptions. Then, we measured the edit distance between similar job descriptions. In our paper, we empirically set the threshold to 20 to distinguish small differences.

As a result, we found less than 1% of the jobs are duplicated. We filtered out these duplicated samples from our training data.

Supplementary Table S3. A concrete example of the company dataset. In our company data, the company information includes company name, establish city, finance stage, establish year, and industry fields.

Company Name	Establish Year	Establish City	Finance Stage	Industry Fields
Company #001	2016	Beijing	Round D and above	Mobile Internet, E-commerce
Company #002	2018	Wuhan	No financing	Consumer life
Company #003	2009	Shanghai	Listed company	Entertainment

Supplementary Table S4. Features Extraction. To form the model input, we extracted features for the contexts and skills from the job posting data.

Feature	Context/Skill	Input Type	Description
Establish City	Company	Discrete	The index of the city where the company was established. Specifically, our training data contains 13 cities of company establishment, mostly contained in our filtered cities of working address.
Finance Stage	Company	Discrete	In the company dataset, finance stages are formed as strings. We merged them into 8 stages, including "no financing required", "no financing", "angel round", "round A", "round B", "round C", "round D and above", and "listed company". Besides, we treated the missing value as another possible value.
Age	Company	Continuous	We subtracted the company establish time from the current time to obtain the company age.
Industry Fields	Company	Continuous	In the company dataset, there are 28 industry fields in total. Since each company can belong to multiple industry fields, we represent this context as a fixed-length vector. Specifically, each element in the vector represents an industry field. One element is 1 if the company belongs to it and otherwise 0.
Experience	Experience	Discrete	The index of the required working experience. Specifically, each job posting requires the recruiter to specify the requirement on the candidates' working experience. The options include "graduate", "0-1 year", "1-3 years", "3-5 years", "5-7 years", "5-10 years", "5-10 years", and "above 10 years". We regarded each of them as a discrete value.
Level Index	Level	Discrete	The index of the level word.
City Index	City	Discrete	In our dataset, each job posting contains the job's working city. We regarded the city index as one discrete input to the model.
City Salary Statistics	City	Continuous	For each city in each time interval, we extracted a feature vector to represent the city's salary status. Specifically, we collected the job postings of this city in the *previous* time interval. Then, we calculated the following statistics of these job postings: 1) the average of the lower bound salary, 2) the lower-quartile of the lower bound salary, 3) the upper-quartile of the lower bound salary, 4) the standard deviation of the lower bound salary, 5) the average of the upper bound salary, 6) the lower-quartile of the upper bound salary, 7) the upper-quartile of the upper bound salary, 8) the standard deviation of the upper bound salary.
Job Benefits	Job Benefits	Continuous	In our data, each job posting contains a paragraph to describe the provided benefits of the job. With the help of domain experts, we extracted 34 kinds of welfare words from the job postings. Then, we matched these words in each job posting. Since each job posting can provide multiple job benefits, we used a fixed-length vector to represent this information. Specifically, each element in the vector represents one welfare word. The value is 1 if the welfare word appears in the job posting and otherwise 0.
Time Index	Time	Discrete	The index of the time interval when the job posting is published.
Skill Index	Skill	Discrete	The index of the skill.
Semantic Embedding	Skill	Continuous	We trained an embedding vector to represent the semantic information of each skill. Specifically, we first transformed each job posting into a skill sequence. Then, we adopted the *word2vec* implementation of *gensim*[52] for training the skill embeddings, which models the neighboring relation between skills to seize the skill semantic.
Skill-Salary Statistics	Skill	Continuous	For each skill in each time interval, we collected the job postings that requires it in the ***previous*** time interval. Then, we extracted a feature vector from these job postings, which contains 1) the number of the jobs, 2) the average of their upper-bound salary, 3) the average of their lower-bound salary, 4) the average of the gaps between their upper-bound salary and lower-bound salary, 5) the standard deviation of the lower-bound salary, 6) the standard deviation of the upper-bound salary, 7) the standard deviation of the gaps between their upper-bound salary and lower-bound salary.

Supplementary Note 3: Level Influence Bias Estimation

Partial Order for Level Influence

To estimate the ratio of biased level influence, we built a general partial ordering set $\mathcal{P}$, where $(\mathrm{lv}_1, \mathrm{lv}_2) \in P$ if $r^{\mathrm{lv}_1} - r^{\mathrm{lv}_2}$ is larger than a threshold. Empirically, we set the threshold to be $\frac{1}{2}|r^{\mathrm{lv}_2}|$ in this work. Next, we built a set $\mathcal{P}^s$ for each specific skill s, where $(\mathrm{lv}_1, \mathrm{lv}_2) \in P$ if $r_s^{\mathrm{lv}_1} - r_s^{\mathrm{lv}_2} > \frac{1}{2}|r_s^{\mathrm{lv}_2}|$ and lv_1 and lv_2 significantly influence the value of this skill.

Biased Level Influence

If the influence estimation of some levels are biased for this skill, there will be inconsistencies between $\mathcal{P}^s$ and $\mathcal{P}$, i.e., $(\mathrm{lv}_1, \mathrm{lv}_2) \in \mathcal{P}^s$ while $(\mathrm{lv}_2, \mathrm{lv}_1) \in \mathcal{P}$. The inconsistency can be eliminated by adjusting $r_s^{\mathrm{lv}_1}$ or $r_s^{\mathrm{lv}_2}$. Notably, a biased level can lead to multiple inconsistent partial ordering relations. For example, if we have an ordering chain in $\mathcal{P}$ as $\mathrm{lv}_1 \succ \mathrm{lv}_2 \succ \mathrm{lv}_3$, if lv_3 has biased large influence estimation and causes $\mathrm{lv}_3 \succ \mathrm{lv}_1 \succ \mathrm{lv}_2$ in $\mathcal{P}^s$, we have two inconsistent partial orderings of $(\mathrm{lv}_3, \mathrm{lv}_1)$ and $(\mathrm{lv}_3, \mathrm{lv}_2)$. Though the two inconsistent pairs involve all the three levels, lv_3 is the only level that causes them.

Bias Ratio Estimation

We aim to estimate the set of causing levels by observing the inconsistency between $\mathcal{P}$ and $\mathcal{P}^s$. To estimate the ratio of biased skill-level observations in our data, we attached each skill-level pair with their appearing frequencies (i.e., the number of their observations). Then, we estimated the minimum number of observations we need to adjust, so that we can change the influence estimation of some levels and make $\mathcal{P}^s$ consistent with $\mathcal{P}$. Notably, if we regard a level as biased, we can adjust its influence to an arbitrary value. Then, for every time we adjust a level, we can eliminate all its related inconsistent partial orderings. Therefore, the problem is equivalent to deleting some levels and make the rest of the levels consistent with $\mathcal{P}$. Using $\mathcal{P}^I$ to denote the set of inconsistent partial orderings, the problem becomes to delete some levels with the minimum total cost (the number of observations), so that for each element $(\mathrm{lv}_1, \mathrm{lv}_2) \in \mathcal{P}^I$, either lv_1 or lv_2 is deleted, which is a classic weighted set cover problem[53]. While the set cover problem is NP-hard and many approximation algorithms have been proposed for it, since we only have a few levels to consider, we designed a definite algorithm to get the accurate answer, as shown in Algorithm 1. We estimated the bias as the ratio between the total number of biased level observations of each skill and the total number of observations of significant skill-level pairs in the dataset.

Algorithm 1 Bias Ratio Estimation

Require: $\mathcal{P}^I$: the inconsistent level pairs

for each level lv **do**
  $O_{lv} \leftarrow \{lv' | (lv, lv') \in \mathcal{P}^I or (lv', lv) \in \mathcal{P}^I\}$;
$Plans \leftarrow \{\emptyset\}$;
$result \leftarrow \infty$;
for each level lv **do**
  for each $plan \in Plans$ **do**
    $count \leftarrow 0$;
    for each $lv' \in O_{lv}$ **do**
      if $lv' \notin plan$ **then**
        $count \leftarrow count + 1$;
    if $count > 0$ **then**
      $nowplan \leftarrow plan \cup \{lv\}$;
      $Plans \leftarrow Plans \cup \{nowplan\}$;
      if $\mathcal{P}^I$ covered by $nowplan$ **then**
        $cost \leftarrow$ involved observations of $nowplan$;
        $result \leftarrow \min(result, cost)$;
return $result$;

Supplementary Experiment 1: Data Analysis

Supplementary Table S5. Statistics of the job posting data. We separately analyzed the IT and designer dataset and show their statistics in the table. It can be observed that our data contains few duplicated job advertisements and has little missing salary information. Indeed, on the website where we have collected our data from, the salary range is required to be specified for each job posting. As a result, there is little missing salary in our dataset.

Name	IT	Designer
Number of job postings	805,184	99,624
Distinct companies	101,426	46,996
Distinct job address	302	233
Distinct job titles	201,771	19,813
Missing Salary Lower-bound	0.015%	0.014%
Missing Salary Upper-bound	0.32%	0.28%
Duplication rate	$< 1\%$	

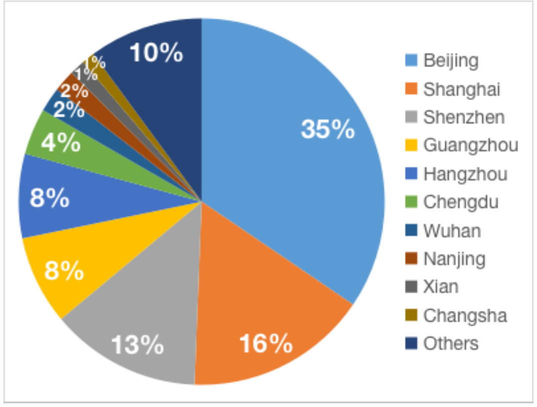

(a) 2016-H2

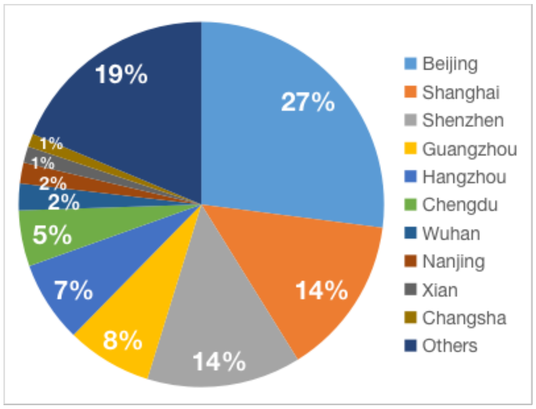

(b) 2017-H1

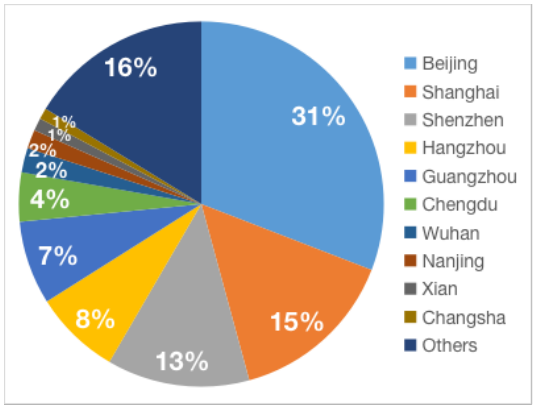

(c) 2017-H2

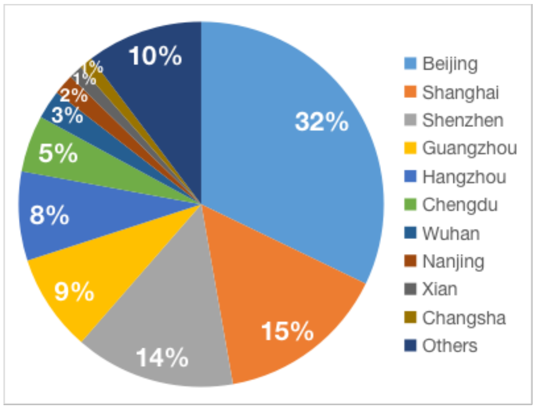

(d) 2018-H1

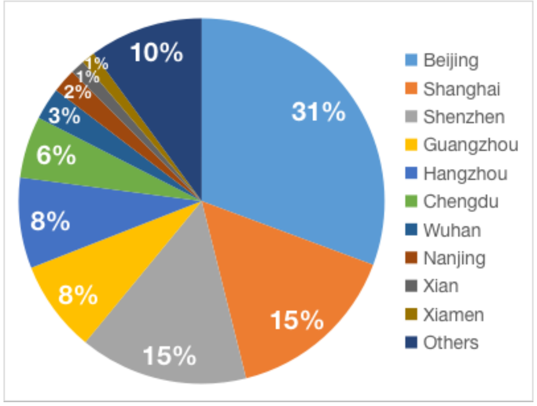

(e) 2018-H2

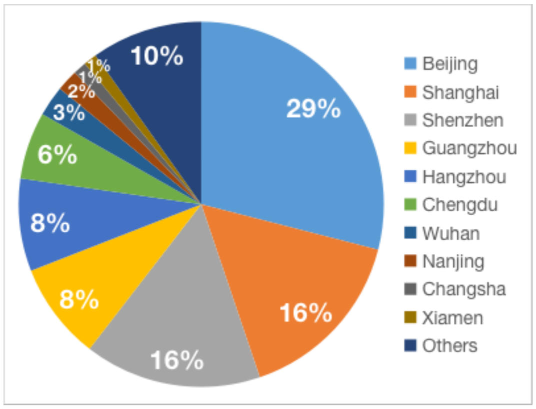

(f) 2019-H1

Supplementary Figure S1. Job posting distribution among cities (IT jobs). The pieplots show the distribution of IT job postings among cities, where H1 denotes the first half of the year and H2 denotes the second half of the year. Here, we use different colors to distinguish different cities. It can be observed that different time periods have similar distributions.

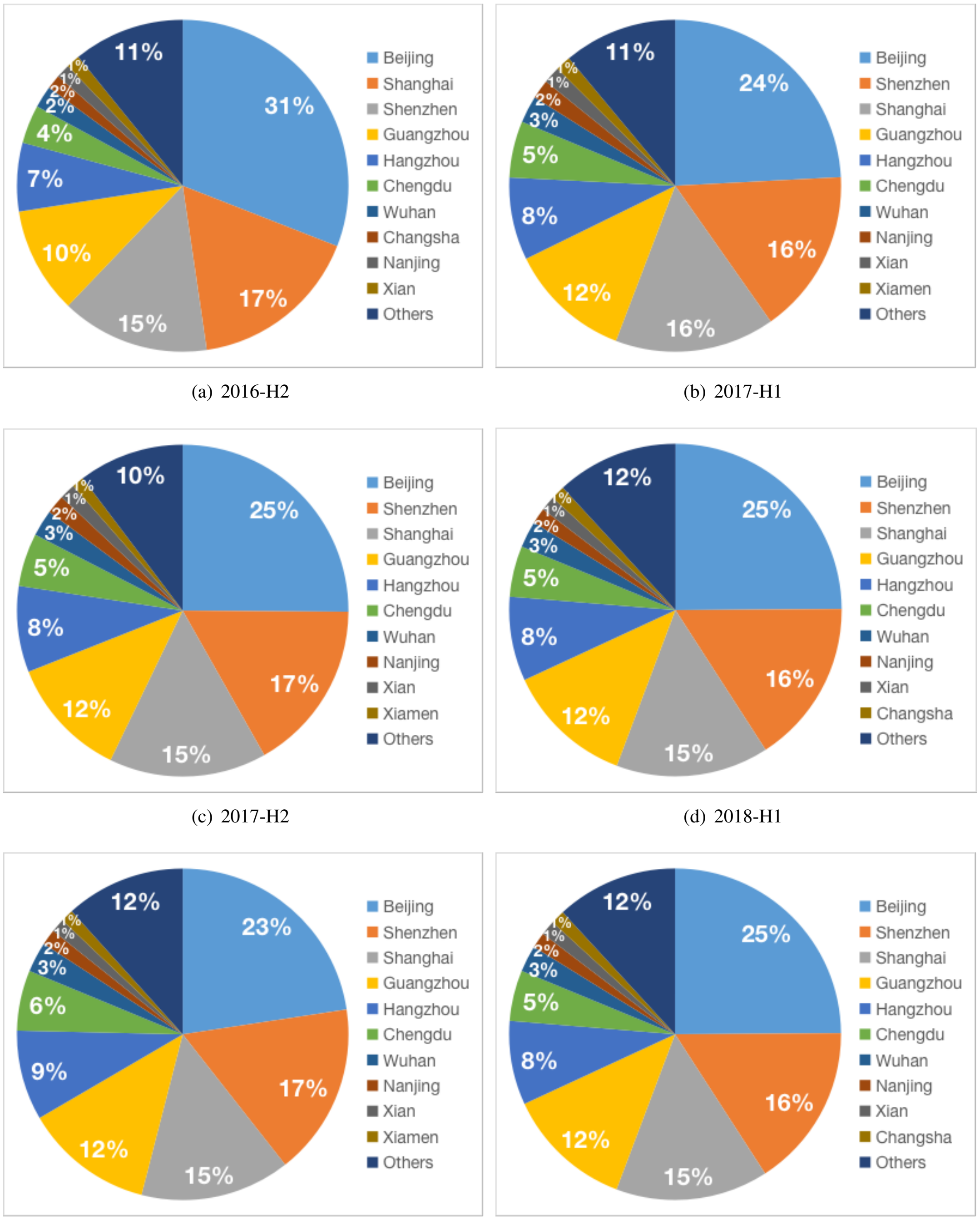

(a) 2016-H2 (b) 2017-H1

(c) 2017-H2 (d) 2018-H1

(e) 2018-H2 (f) 2019-H1

Supplementary Figure S2. Job posting distribution among cities (designer jobs). The pieplots show the distribution of designer job postings among cities, where H1 denotes the first half of the year and H2 denotes the second half of the year. Here, we use different colors to distinguish different cities. It can be observed that different time periods have similar distributions.

Supplementary Table S6. The number of job postings in different cities. This table lists the 30 cities that have the most job postings in the IT dataset and the designer dataset, respectively.

Data	City	Jobs	City	Jobs	City	Jobs	City	Jobs	City	Jobs
IT	Beijing	255,651	Wuhan	20,808	Chongqing	6,747	Qingdao	2,955	Ningbo	1,597
	Shanghai	124,253	Nanjing	15,694	Zhengzhou	6,411	Jinan	2,944	Shenyang	1,238
	Shenzhen	116,498	Xian	10,293	Hefei	4,634	Zhuhai	2,920	Shijiazhuang	1,195
	Guangzhou	66,541	Changsha	9,422	Tianjin	4,412	Dongguan	2,479	Nanchang	1,144
	Hangzhou	64,074	Xiamen	8,883	Fuzhou	3,094	Dalian	2,180	Nanning	1,087
	Chengdu	41,189	Suzhou	8,074	Foshan	3,067	Wuxi	1,807	Kunming	1,077
Designer	Beijing	24,741	Wuhan	2,645	Zhengzhou	916	Zhuhai	430	Dalian	206
	Shenzhen	16,141	Nanjing	1,573	Chongqing	784	Qingdao	394	Nanchang	183
	Shanghai	15,103	Xian	1,390	Tianjin	613	Jinan	377	Shijiazhuang	164
	Guangzhou	11,993	Xiamen	1,333	Foshan	596	Fuzhou	376	Kunming	161
	Hangzhou	8,136	Changsha	1,271	Hefei	519	Ningbo	241	Nanning	153
	Chengdu	5,202	Suzhou	980	Dongguan	506	Wuxi	221	Shenyang	151

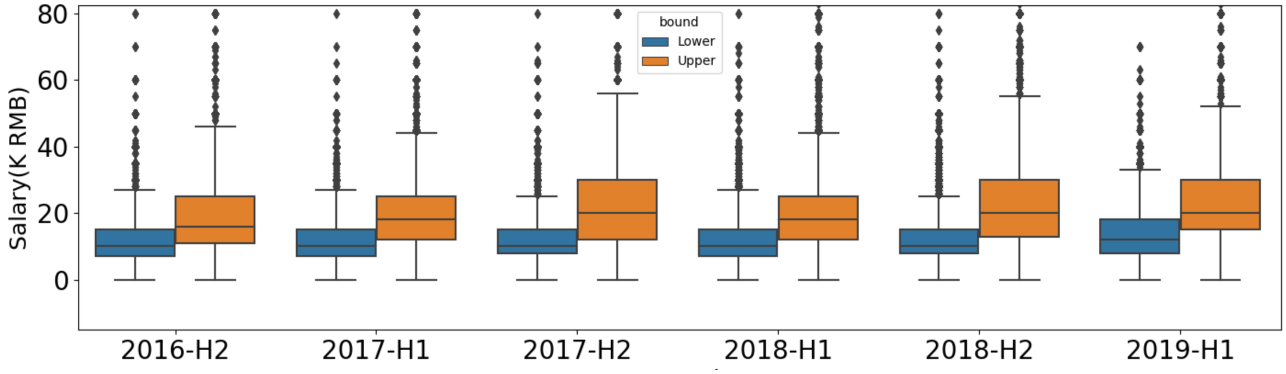

(a) IT

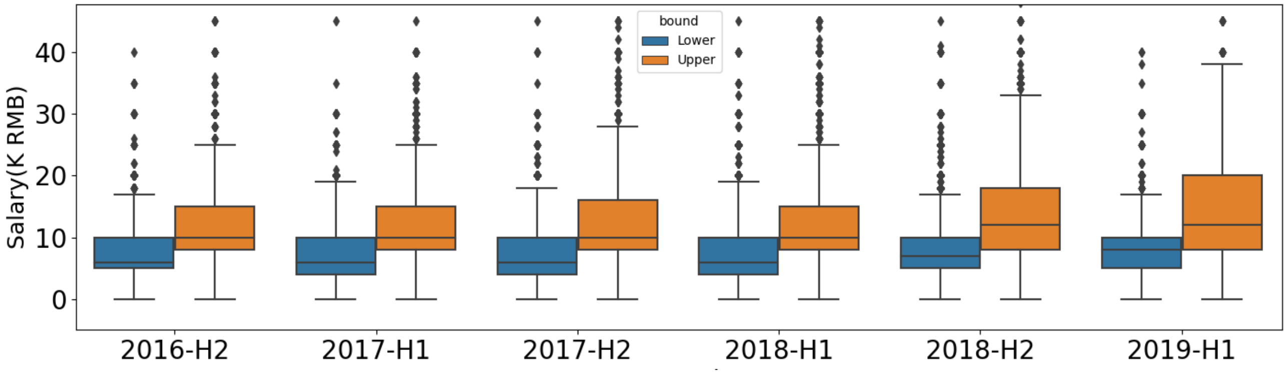

(b) Designer

Supplementary Figure S3. Boxplot of salary distribution. This figure shows the boxplot of the salary distribution in the IT dataset and the designer dataset, at different time periods. The box shows the quartiles of the dataset. The whiskers extend to show the rest of the distribution except for outliers. Specifically, we regard the samples that outside 1.5 times interquartile range (IQR) above the upper quartile or below the lower quartile as outliers. We have used all the instances in the corresponding time period for the estimation. The detailed sample size information can be found in Supplementary Table S22. Here, we use different colors to indicate different bounds.

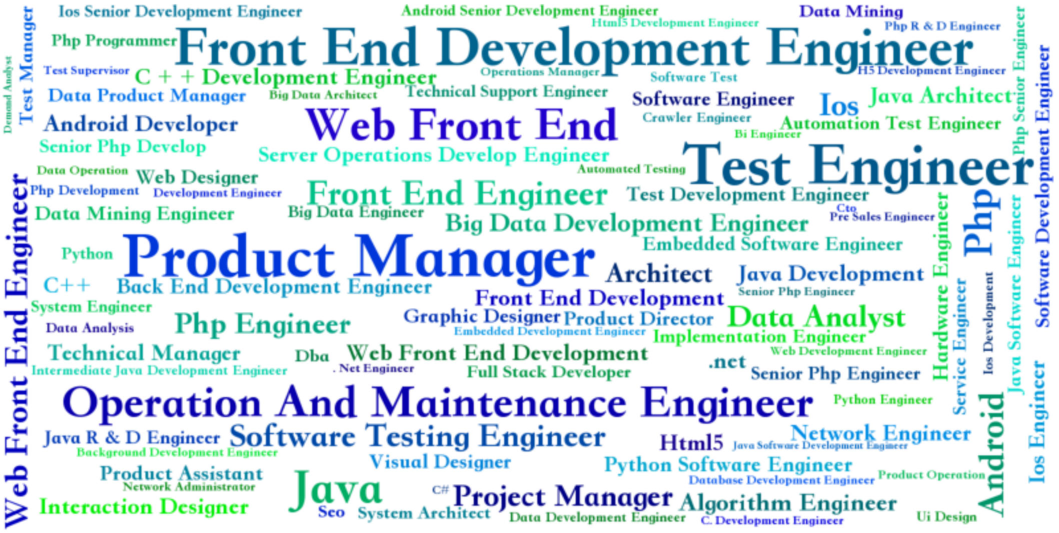

(a) IT

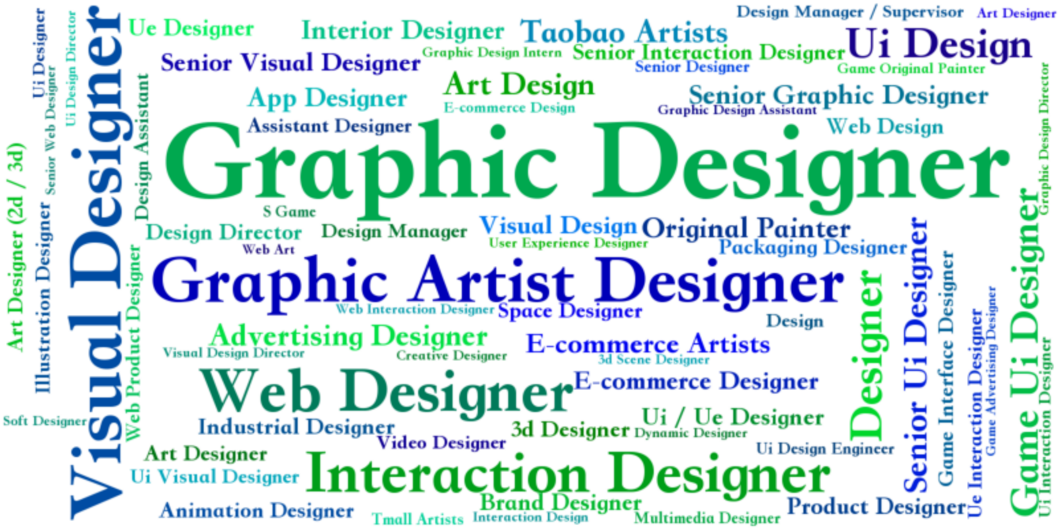

(b) Designer

Supplementary Figure S4. Wordcloud of job titles. In our dataset, each job posting has a customized job title that briefly specifies the occupation. The wordclouds here show the number of job titles in the IT datasets and the designer dataset, respectively. It can be observed that our datasets cover various kinds of jobs.

Supplementary Experiment 2: Parameter Experiments

Supplementary Table S7. Parameter Experiment. To show our model's parameter insensitivity and robustness, we adjusted the model parameters and evaluated performance for different settings of the parameters. Specifically, for each set of experiments, we adjusted one of the parameters and set the others as default. Then, we conducted 10 times of holdout validation on the IT-dataset with a ratio of 4:1. The results of RMSE and MAE for the test set are listed here, shown as *mean* ± *standard deviation*. It can be observed that our model is robust and insensitive to the change of the model parameters. Therefore, our model can be easily applied without specially tuning the parameters.

Parameter	Value	Lower		Upper	
		RMSE	MAE	RMSE	MAE
Inf/Imp MLP (depth)	1	4.446±0.079	3.251±0.061	7.671±0.095	5.604±0.074
	2	4.419±0.047	3.229±0.037	7.632±0.073	5.577±0.059
	3	4.435±0.061	3.244±0.048	7.686±0.086	5.627±0.060
	4	4.447±0.054	3.248±0.045	7.652±0.052	5.587±0.041
	5	4.470±0.072	3.264±0.060	7.702±0.086	5.627±0.069
Inf/Imp MLP (hidden)	8	4.440±0.047	3.242±0.038	7.660±0.077	5.591±0.059
	16	4.435±0.061	3.244±0.048	7.686±0.086	5.627±0.060
	24	4.460±0.048	3.261±0.043	7.673±0.051	5.607±0.042
	32	4.520±0.048	3.306±0.040	7.742±0.074	5.658±0.060
CSVN MLP share (depth)	1	4.417±0.054	3.233±0.046	7.620±0.078	5.573±0.059
	2	4.444±0.051	3.250±0.038	7.640±0.052	5.584±0.039
	3	4.435±0.061	3.244±0.048	7.686±0.086	5.627±0.060
	4	4.464±0.074	3.264±0.060	7.709±0.083	5.630±0.070
CSVN MLP share (hidden)	16	4.469±0.032	3.273±0.027	7.737±0.045	5.665±0.036
	32	4.449±0.057	3.250±0.046	7.711±0.064	5.638±0.049
	48	4.435±0.061	3.244±0.048	7.686±0.086	5.627±0.060
	64	4.430±0.062	3.238±0.050	7.657±0.054	5.594±0.045
	96	4.450±0.055	3.253±0.046	7.639±0.099	5.580±0.080
GCN (depth)	1	4.476±0.640	3.274±0.054	7.716±0.081	5.641±0.066
	2	4.435±0.061	3.244±0.048	7.686±0.086	5.627±0.060
	3	4.461±0.057	3.261±0.046	7.705±0.071	5.633±0.053
GCN (hidden)	8	4.418±0.043	3.226±0.031	7.640±0.091	5.579±0.066
	16	4.435±0.061	3.244±0.048	7.648±0.086	5.627±0.060
	24	4.457±0.054	3.260±0.045	7.695±0.060	5.623±0.049
CSVN MLP individual (depth)	1	4.506±0.052	3.296±0.041	7.757±0.056	5.675±0.046
	2	4.445±0.055	3.250±0.045	7.666±0.049	5.597±0.041
	3	4.435±0.061	3.244±0.048	7.686±0.086	5.627±0.060
	4	4.470±0.077	3.276±0.057	7.671±0.076	5.616±0.061
CSVN MLP individual (hidden)	8	4.446±0.060	3.249±0.049	7.673±0.078	5.604±0.063
	16	4.435±0.061	3.244±0.048	7.686±0.086	5.627±0.060
	24	4.450±0.073	3.252±0.057	7.696±0.097	5.621±0.074
Embedding	8	4.481±0.046	3.274±0.038	7.756±0.071	5.672±0.057
	16	4.435±0.061	3.244±0.048	7.686±0.086	5.627±0.060
	24	4.445±0.067	3.250±0.053	7.633±0.088	5.578±0.072
	32	4.402±0.054	3.218±0.042	7.580±0.054	5.539±0.044

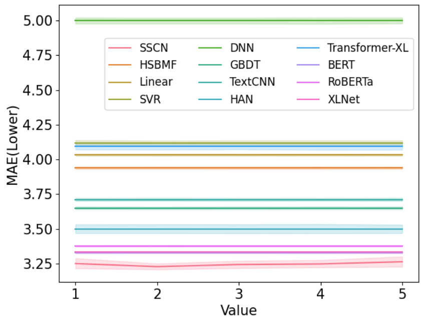

(a) Inf/Imp MLP(depth)

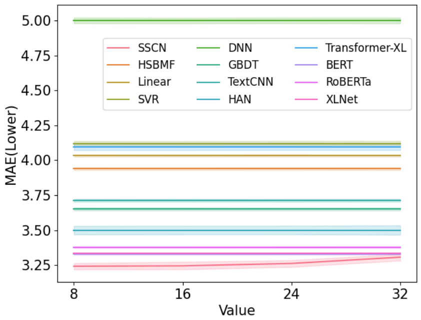

(b) Inf/Imp MLP(hidden)

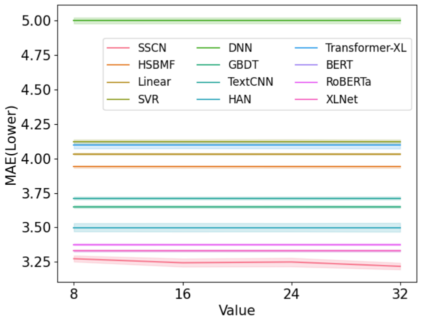

(c) Embedding Size

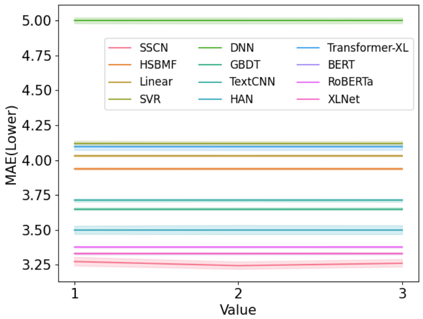

(d) GCN(depth)

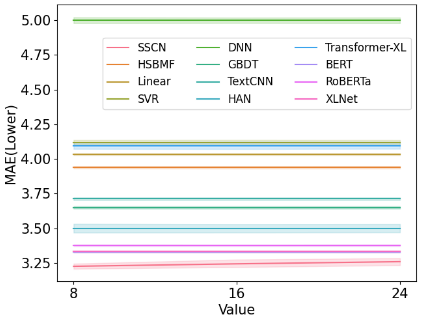

(e) GCN(hidden)

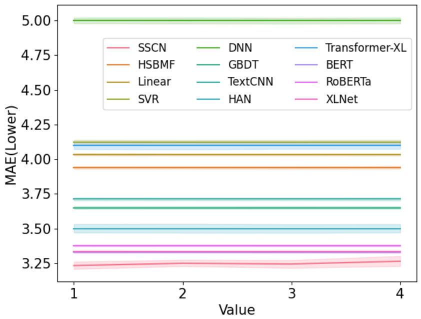

(f) CSVN MLP share (depth)

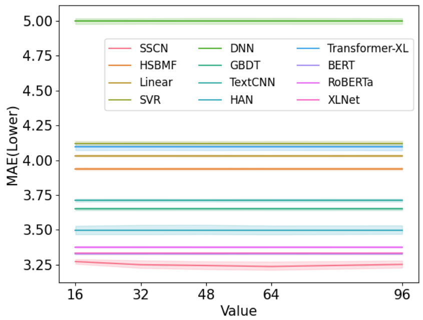

(g) CSVN MLP share (hidden)

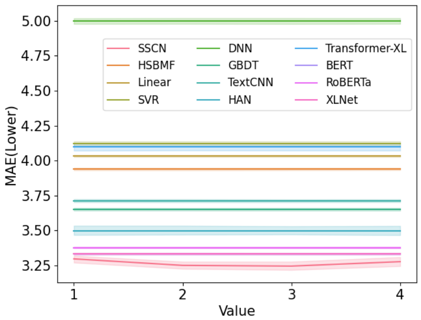

(h) CSVN MLP individual (depth)

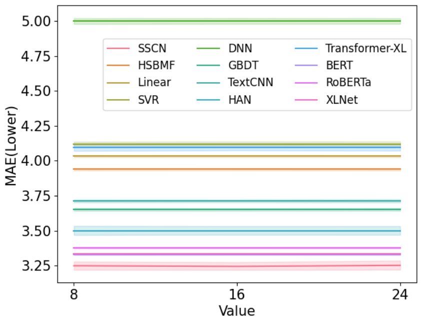

(i) CSVN MLP individual (hidden)

Supplementary Figure S5. Parameter Experiment. To show our model's parameter insensitivity and robustness, we adjusted the model parameters and evaluated performance for different settings of the parameters. Specifically, for each set of experiments, we adjusted one of the parameters and set the others as default. Then, we conducted 10 times of holdout validation on the IT-dataset with a ratio of 4:1. In this figure, we show the MAE on lower-bound salary for different parameters. Specifically, data are presented as mean values ± standard deviation (SD). Here, we use different colors to distinguish the performance of different models. To more explicitly show the performance, we also show in the figure the performance of the baselines. It can be observed that our model is robust and insensitive to the model parameter, thus can be applied easily without much effort on parameter tuning.

Supplementary Experiment 3: Experiment on the Designer Dataset

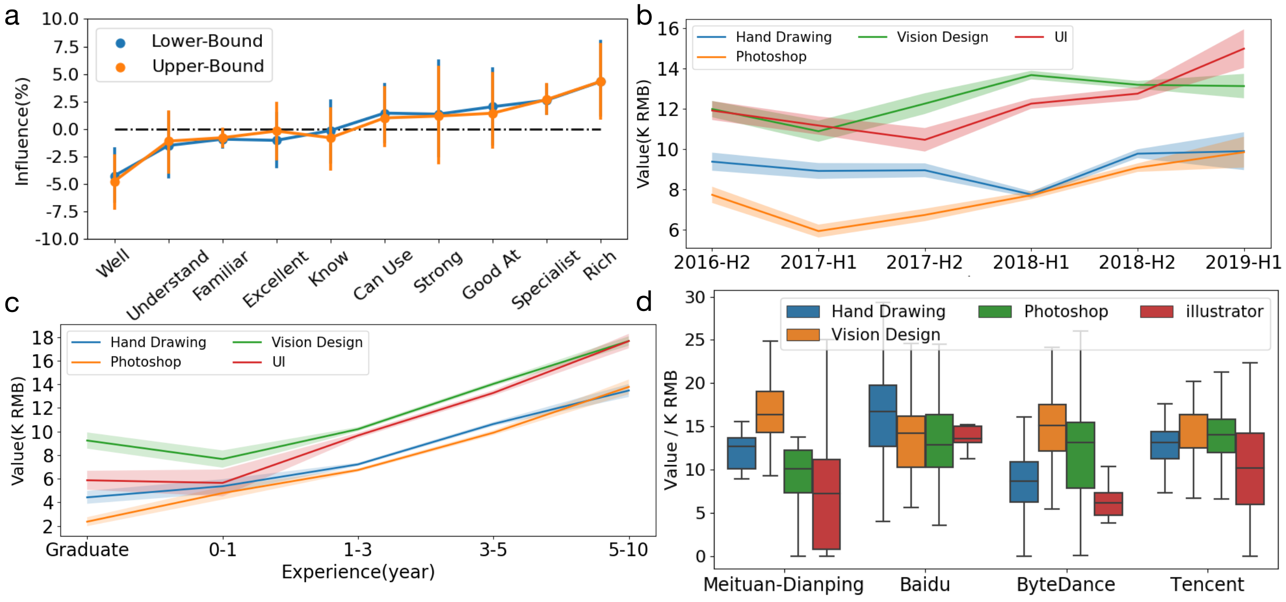

Supplementary Figure S6. Skill valuation concerning different job contexts (designer-related jobs). (a) Similar to the IT dataset, we calculated the influence of level lv as $r_s^{\text{lv}} = \frac{\sum_{i,j} \mathbb{1}\{\text{lv}_j^{(i)}=\text{lv}\}(v_j^{(i)} - v_{s_j^{(i)}})/v_{s_j^{(i)}}}{\sum_{i,j} \mathbb{1}\{\text{lv}_j^{(i)}=\text{lv}\}}$, where $v_{s_j^{(i)}}$ denotes the averaged value of skill $s_j^{(i)}$. The figure also shows the 95% confidence interval, where data are presented as mean values $\pm$ CI. We used all the skill-level pair instances of each level for the estimation. The detailed sample size information can be found in Supplementary Table S11. Different colors indicate the influence on different bounds. We dropped level words that rarely appear in the designer dataset. **(b)** CSVN assigns the skills with temporal embedding vectors to catch their dynamic changes. This figure shows the average value of some skills at different time intervals. The shadow shows the 95% confidence interval, where data are presented as mean values $\pm$ CI. We used all the instances of the corresponding skill-time pairs for the estimation. The detailed sample size information can be found in Supplementary Table S12.Different colors indicate different skills. It can be observed that the value of designer skills are relatively stable over time. **(c)** The average value of some randomly selected skills under different lengths of working experiences. The shadow shows the 95% confidence interval, where data are presented as mean values $\pm$ CI. We used all the instances of the corresponding skill-experience pairs for the estimation. The detailed sample size information can be found in Supplementary Table S13. We use different colors to indicate different skills. Since the designer dataset has a relatively smaller size, the experience of above 10 years rarely appears in the dataset. Therefore, we dropped working experience above 10 years in this figure. It can be observed that, consistent with our previous findings, a longer experience leads to a higher skill value. **(d)** The value distribution of some popular skills in 4 famous Chinese Internet companies. The box shows the quartiles of the dataset. The whiskers extend to show the rest of the distribution except for outliers. Specifically, as a common practice, we regarded the samples outside 1.5 times interquartile range (IQR) above the upper quartile or below the lower quartile as outliers. We have used all the instances containing the corresponding skill-company pairs for the estimation. The detailed sample size information can be found in Supplementary Table S13. Different colors indicate different skills. Compared with the IT-dataset, since JD.com rarely appears in the designer dataset, we dropped it from the figure. It can be observed that these companies have different preferences for these designer skills.

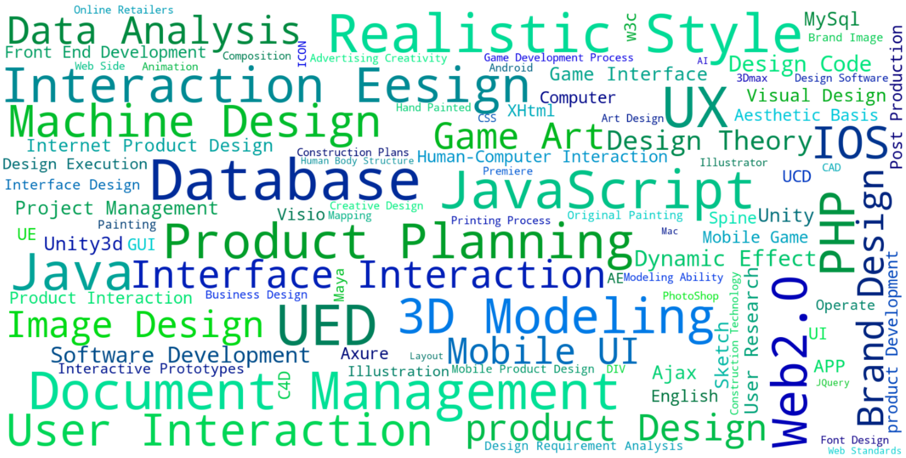

(a) Value

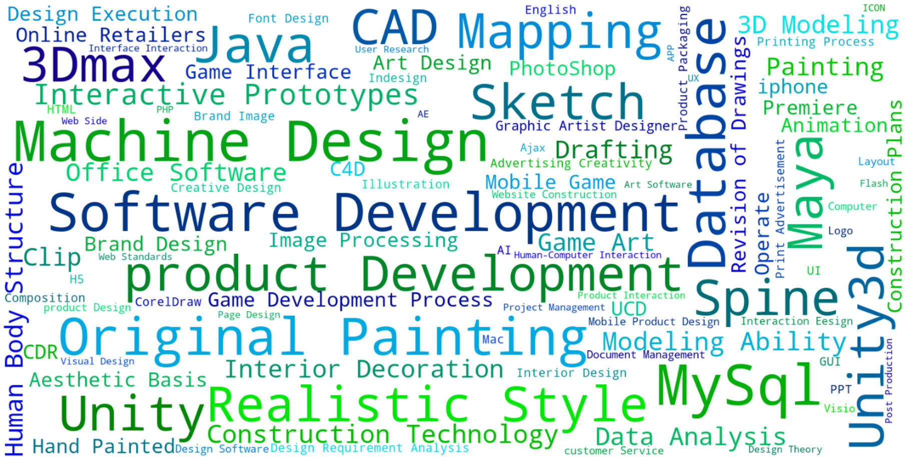

(b) Domination

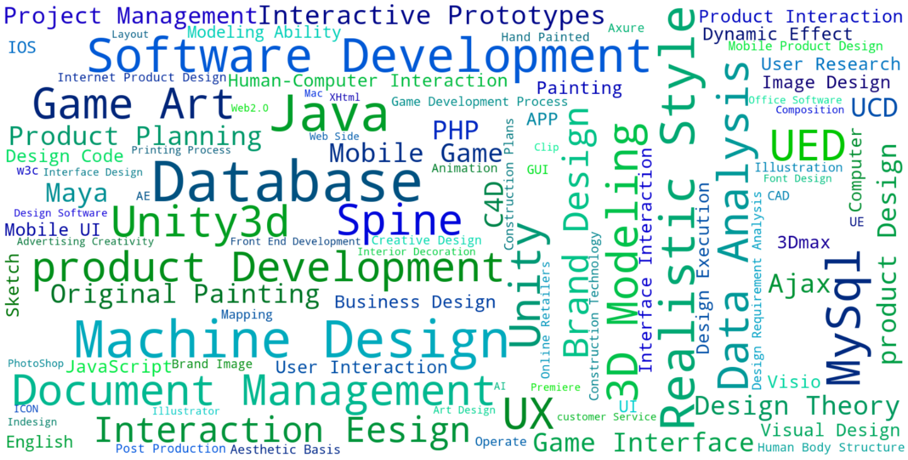

(c) Contribution

Supplementary Figure S7. The wordclouds for designer skills. The wordclouds show the value, domination and contribution of the designer skills.

Supplementary Table S8. Performance evaluation on salary prediction (designer dataset). We validated our model's performance on salary prediction for the designer job postings. Specifically, 10 times of holdout validation were repeated on each model. At each time, we randomly split the data for training and testing with a ratio of 4:1. The results of RMSE and MAE are listed in the form of *mean* $\pm$ *standard deviation*. Bold formatting indicates the best performance among all these models. It can be observed that SSCN outperforms the baseline models on this dataset, showing that our model can be generalized to various kinds of occupations.

Model	Lower		Upper	
	RMSE	MAE	RMSE	MAE
SVM	4.340±0.114	3.181±0.072	7.434±0.173	5.454±0.094
LR	4.176±0.068	4.176±0.068	7.326±0.132	5.418±0.065
GBDT	3.525±0.066	2.580±0.036	6.336±0.089	4.700±0.060
DNN	5.241±0.067	4.068±0.063	9.183±0.188	7.210±0.098
HSBMF	4.612±0.025	3.371±0.021	7.800±0.101	5.763±0.075
TextCNN	4.290±0.095	3.164±0.066	7.470±0.153	5.533±0.125
HAN	4.069±0.127	3.024±0.160	7.128±0.192	5.331±0.226
Transformer-XL	4.844±0.039	3.648±0.042	8.499±0.080	6.492±0.046
BERT	3.777±0.075	2.638±0.058	6.896±0.335	4.965±0.324
RoBERTa	3.843±0.096	2.726±0.053	6.985±0.123	5.115±0.109
XLNet	3.701±0.067	2.641±0.041	6.699±0.137	4.767±0.074
CSVN + Mean	4.615±0.063	3.39±0.048	7.690±0.177	5.637±0.113
SSCN (Independ)	3.841±0.143	2.766±0.102	6.525±0.087	4.692±0.068
SSCN	**3.163±0.064**	**2.312±0.047**	**5.307±0.110**	**3.960±0.075**

Supplementary Experimental Results

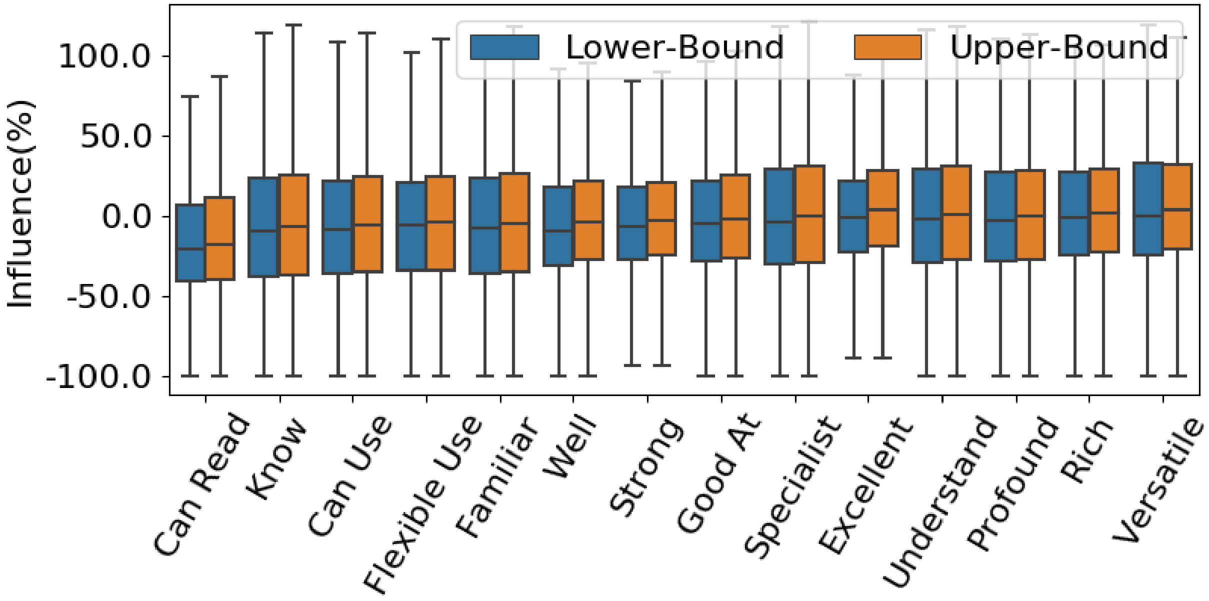

(a) IT

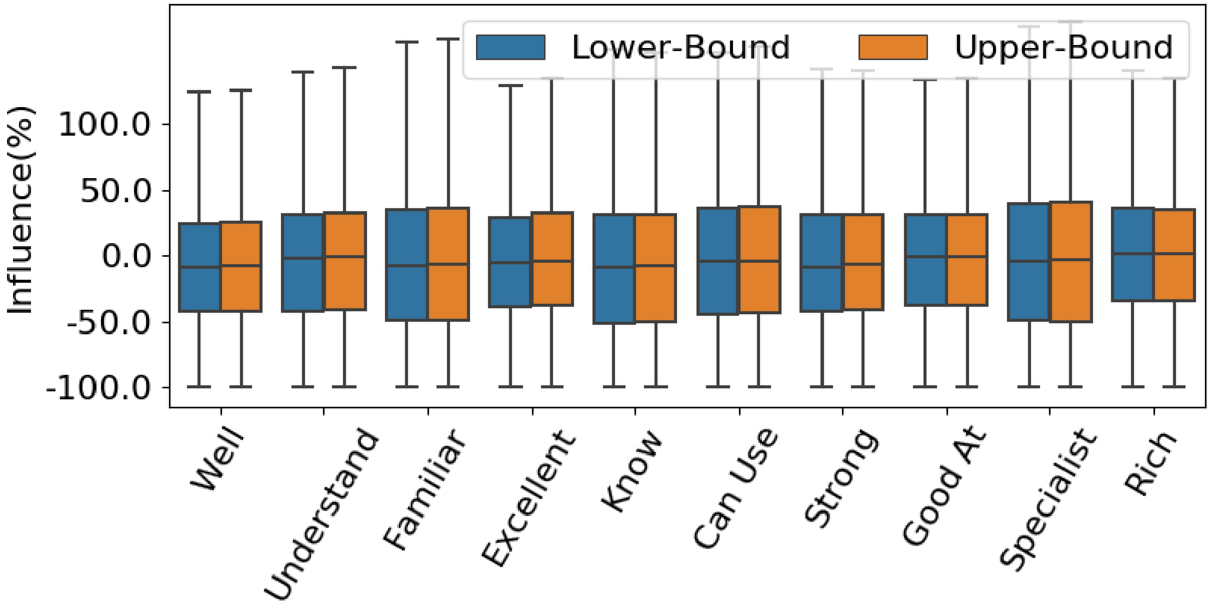

(b) Designer

Supplementary Figure S8. The boxplots for level influence on the IT dataset and the designer dataset. For each skill i required by each job posting j, we computed the increase ratio of its value $v_j^{(i)}$ to the skill's average value $v_{s_j^{(i)}}$, formulated as $(v_j^{(i)} - v_{s_j^{(i)}})/v_{s_j^{(i)}}$. Then, we show the distribution of this ratio with the boxplots, distinguishing the levels that influence them. The box shows the quartiles of the dataset. The whiskers extend to show the rest of the distribution except for outliers. As a common practice, we regarded the samples outside 1.5 times interquartile range (IQR) above the upper quartile or below the lower quartile as outliers. We used all the skill-level pair instances involving each level for the estimation. The detailed information on sample size can be found in Supplementary Table S10. Different colors indicate different bounds. It can be observed that the variance is large, for there are other influencing factors of skill value. However, the levels still cause general shifts in the distribution.

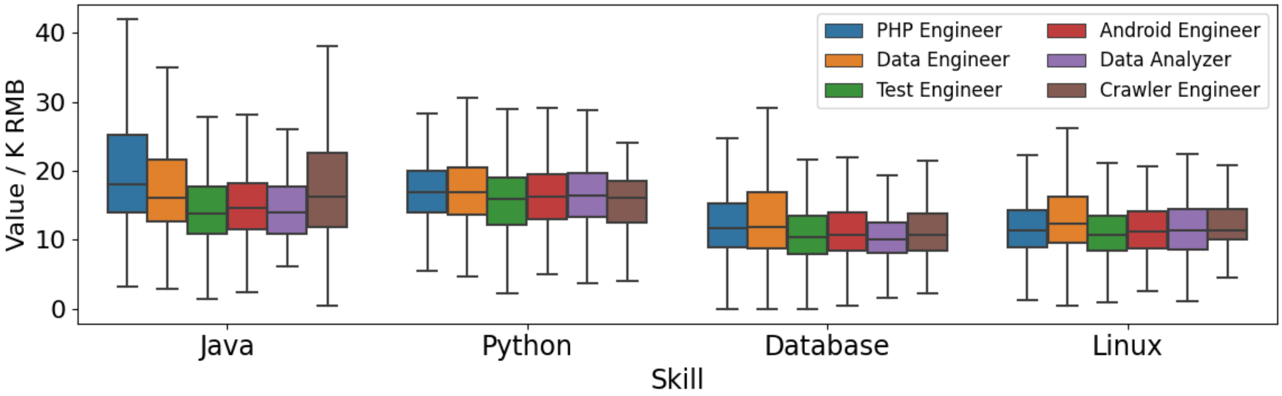

Supplementary Figure S9. Skill value distribution in different job positions. The boxplot shows the value distribution of 4 common skills in 6 frequently-appearing job positions in the designer dataset. The box shows the quartiles of the dataset. The whiskers extend to show the rest of the distribution except for outliers. We regarded the samples outside 1.5 times interquartile range (IQR) above the upper quartile or below the lower quartile as outliers. We used all the skill instances in job postings that belong to each job position for the estimation. The detailed information on sample size can be found in Supplementary Table S23. Different colors indicate different skills. It can be observed that the distributions only slightly differ on different occupations, which is caused by the varied context distribution of these occupations. This result proves our model to achieve general skill value assessment, which is shared across the job market.

Supplementary Table S9. Skill value rank in different cities. We ranked several common programming skills by their value in 6 big cities, including Beijing, Shanghai, Shenzhen, Hangzhou, Guangzhou, and Chengdu. In this table, we also show the number of job postings in each city that involve these skills. It can be observed that the rank is generally similar in different cities. Nevertheless, there are some differences that implies the traits of the cities. For example, *c/c++* ranks high in Shenzhen, which is different from the other cities.

Rank	Beijing			Shanghai			Shenzhen		
	Skill	Count	Value	Skill	Count	Value	Skill	Count	Value
1	golang	5,906	26.88	golang	1,865	25.42	scala	492	24.36
2	java	33,481	21.36	scala	772	21.86	golang	1,293	22.96
3	scala	2,163	21.00	java	12,808	20.26	java	8,492	18.21
4	python	26,594	20.78	python	8,719	17.86	R	850	16.68
5	javascript	14,033	17.28	javascript	6,906	16.82	python	6,491	15.99
6	objective-c	2,024	16.33	R	1,439	16.37	c/c++	3,270	15.52
7	php	9,735	15.56	objective-c	653	15.07	javascript	4,713	14.44
8	R	3,783	15.36	php	2,615	14.81	perl	941	14.42
9	ruby	1,973	15.15	perl	1,208	14.21	objective-c	437	14.10
10	perl	2,783	15.12	ruby	746	13.57	ruby	451	13.14
11	sql	15,365	13.60	sql	7,366	13.07	php	2,122	13.05
12	c/c++	9,641	12.91	c/c++	2,498	11.28	sql	4,737	12.46
13	c#	1,379	10.35	c#	1,039	9.38	c#	613	11.11

Rank	Hangzhou			Guangzhou			Chengdu		
	Skill	Count	Value	Skill	Count	Value	Skill	Count	Value
1	scala	352	22.58	scala	123	24.46	golang	258	18.86
2	golang	587	21.34	golang	359	18.65	scala	136	16.57
3	java	7,055	18.25	java	3,451	16.70	java	2,176	15.09
4	python	3,741	15.96	R	244	13.28	python	1,399	14.55
5	javascript	2,516	15.05	python	2,328	13.11	perl	137	13.39
6	R	559	14.23	javascript	2,221	12.90	javascript	1,090	12.97
7	perl	569	13.85	objective-c	207	11.97	R	289	11.66
8	objective-c	273	13.26	perl	283	11.96	php	349	10.38
9	php	1,143	12.43	php	902	11.83	c/c++	459	10.21
10	ruby	369	11.80	ruby	126	11.71	objective-c	84	9.60
11	sql	2,778	11.63	sql	1,926	11.13	ruby	176	9.26
12	c/c++	1,489	10.69	c/c++	1,038	10.72	sql	1,047	8.99
13	c#	285	7.69	c#	335	7.79	c#	238	7.48

Supplementary Tables: Numerical Statistics of the Original Figures

Supplementary Table S10. Statistics of value increase ratio distribution under different levels (IT dataset). This table shows the mean (and the 95% confidence interval), lower quartile, median and upper quartile of the value increase ratio (i.e., $(v_j^{(i)} - v_{s_j^{(i)}})/v_{s_j^{(i)}}$) on the IT dataset. We have used all the instances of the corresponding skill-level pairs for the estimation. The sample size of each level is listed below the level word. Since there are other influential factors for skill value, the ratio distribution is large. However, the levels cause general shifts in the value distribution.

Level	Bound	Mean(%)	Lower Quartile(%)	Median(%)	Upper Quartile(%)	Lower CI (%)	Upper CI (%)
Can Read<br>521	Lower-Bound	-10.67	-40.31	-20.31	6.84	-15.29	-6.05
	Upper-Bound	-12.03	-39.66	-18.05	11.11	-15.84	-8.22
Can Use<br>90,377	Lower-Bound	-1.66	-35.47	-8.46	22.26	-2.03	-1.29
	Upper-Bound	-1.84	-34.87	-5.76	24.69	-2.20	-1.48
Excellent<br>12,019	Lower-Bound	4.00	-22.35	-0.35	21.95	3.20	4.81
	Upper-Bound	6.24	-18.55	4.37	28.33	5.51	6.98
Familiar<br>1,145,439	Lower-Bound	-0.70	-35.21	-7.59	23.87	-0.81	-0.59
	Upper-Bound	-0.74	-34.62	-4.84	26.31	-0.85	-0.64
Flexible Use<br>1,153	Lower-Bound	-1.06	-33.79	-5.57	21.00	-4.36	2.24
	Upper-Bound	-0.25	-33.56	-3.50	24.57	-3.59	3.08
Good At<br>8,693	Lower-Bound	2.09	-27.83	-4.38	22.17	1.00	3.19
	Upper-Bound	2.51	-26.19	-1.25	25.43	1.48	3.54
Know<br>112,964	Lower-Bound	-2.18	-37.46	-8.94	23.35	-2.52	-1.83
	Upper-Bound	-2.34	-37.08	-6.55	25.20	-2.68	-2.01
Profound<br>8,565	Lower-Bound	4.15	-28.33	-2.90	27.29	2.99	5.32
	Upper-Bound	3.92	-27.63	-0.12	28.95	2.83	5.00
Rich<br>16,879	Lower-Bound	6.74	-24.75	-1.18	27.78	5.87	7.60
	Upper-Bound	6.52	-22.54	2.30	29.03	5.72	7.33
Specialist<br>234,521	Lower-Bound	4.50	-30.32	-3.14	29.08	4.26	4.74
	Upper-Bound	4.31	-28.92	0.01	31.20	4.08	4.54
Strong<br>8,428	Lower-Bound	-0.50	-27.04	-6.50	17.69	-1.49	0.49
	Upper-Bound	-0.12	-24.79	-2.52	20.91	-0.99	0.75
Understand<br>40,289	Lower-Bound	5.14	-28.62	-1.83	29.39	4.57	5.70
	Upper-Bound	4.95	-27.52	0.90	30.92	4.41	5.50
Versatile<br>4,026	Lower-Bound	9.65	-24.33	0.18	33.19	7.95	11.34
	Upper-Bound	8.47	-20.86	3.85	32.31	6.92	10.02
Well<br>44,151	Lower-Bound	-1.14	-30.64	-8.92	18.28	-1.58	-0.70
	Upper-Bound	-0.22	-27.43	-3.57	21.98	-0.62	0.17

Supplementary Table S11. Statistics of value increase ratio distribution under different levels (designer dataset). This table shows the mean (and the 95% confidence interval), lower quartile, median, and upper quartile of the value increase ratio (i.e., $(v_j^{(i)} - v_{s_j^{(i)}})/v_{s_j^{(i)}}$) on the designer dataset. We have used all the instances of the corresponding skill-level pairs for the estimation. The sample size of each level is listed below the level word. Since there are other influential factors for skill value, the ratio distribution is large. However, the levels cause general shifts in the value distribution.

Level	Bound	Mean(%)	Lower Quartile (%)	Median(%)	Upper Quartile (%)	Lower CI (%)	Upper CI (%)
Can Use<br>2,888	Lower-Bound	1.45	-43.84	-4.36	35.93	-1.15	4.05
	Upper-Bound	1.01	-43.66	-3.95	37.62	-1.55	3.57
Excellent<br>2,155	Lower-Bound	-1.02	-38.63	-5.82	28.77	-3.55	1.50
	Upper-Bound	-0.18	-37.52	-3.95	32.16	-2.71	2.36
Familiar<br>35,970	Lower-Bound	-0.92	-49.30	-7.14	35.28	-1.68	-0.17
	Upper-Bound	-0.79	-49.15	-6.13	36.26	-1.53	-0.04
Good At<br>1,560	Lower-Bound	2.03	-37.01	-0.63	31.66	-1.36	5.42
	Upper-Bound	1.44	-37.73	-0.19	31.21	-1.94	4.81
Know<br>3,162	Lower-Bound	-0.14	-50.94	-8.97	31.95	-3.03	2.76
	Upper-Bound	-0.79	-50.04	-8.14	31.59	-3.59	2.01
Rich<br>1,686	Lower-Bound	4.31	-34.57	1.64	35.54	1.05	7.56
	Upper-Bound	4.33	-33.60	1.95	35.38	1.05	7.60
Specialist<br>13,268	Lower-Bound	2.62	-49.45	-4.25	39.95	1.29	3.94
	Upper-Bound	2.67	-49.68	-3.36	41.12	1.37	3.98
Strong<br>1,064	Lower-Bound	1.36	-41.51	-8.56	31.78	-2.92	5.63
	Upper-Bound	1.18	-40.62	-6.68	31.97	-3.03	5.40
Understand<br>1,788	Lower-Bound	-1.50	-41.58	-2.35	31.46	-4.35	1.35
	Upper-Bound	-1.10	-40.54	-1.10	32.89	-3.89	1.69
Well<br>2,459	Lower-Bound	-4.25	-42.31	-8.45	24.29	-6.68	-1.82
	Upper-Bound	-4.74	-42.15	-7.90	25.11	-7.12	-2.37

Supplementary Table S12. Skill value with time-awareness. This table lists the value of different skills in different time periods, formed as *average (95% confidence interval)*. The sample size of each skill-time pair is listed below the statistics.

Skill	2016-H2	2017-H1	2017-H2	2018-H1	2018-H2	2019-H1
Architecture	21.8(21.6, 22.1)	23.0(22.7, 23.2)	27.9(27.6, 28.1)	26.1(26.0, 26.3)	28.8(28.6, 28.9)	27.6(27.2, 28.0)
	5,864	7,080	8,493	19,730	20,438	3,497
Recommender System	26.1(25.1, 27.0)	23.1(22.4, 23.7)	23.8(22.8, 24.8)	26.9(26.6, 27.1)	23.1(22.8, 23.4)	19.1(18.6, 19.7)
	320	452	672	1,416	1,542	369
c/c++	10.1(9.6, 10.7)	15.5(15.2, 15.8)	12.2(11.9, 12.4)	11.5(11.3, 11.7)	14.0(13.8, 14.2)	10.0(9.6, 10.4)
	2,050	2,050	2,632	5,590	6,039	1,199
golang	27.0(25.8, 28.1)	21.9(21.6, 22.3)	18.3(18.0, 18.6)	25.4(25.2, 25.6)	28.2(28.0, 28.4)	20.8(20.1, 21.4)
	472	826	1,330	3,386	3,958	740
Algorithm	17.5(17.3, 17.8)	18.8(18.5, 19.1)	24.5(24.3, 24.8)	21.1(20.9, 21.2)	21.7(21.6, 21.8)	18.5(18.2, 18.8)
	3,175	3,957	5,982	12,883	14,098	2,974
Hand Drawing	9.4(8.9, 9.8)	8.9(8.5, 9.3)	9(8.6, 9.3)	7.8(7.6, 7.9)	9.8(9.6, 10.0)	9.9(9.0, 10.8)
	430	505	633	1,678	1,764	269
Photoshop	7.7(7.3, 8.1)	5.9(5.6, 6.3)	6.7(6.4, 7.0)	7.7(7.5, 7.9)	9.1(8.9, 9.3)	9.9(9.1, 10.6)
	1,035	1,167	1,263	3,665	3,547	514
Vision Design	12.0(11.6, 12.4)	10.9(10.4, 11.4)	12.3(11.7, 12.8)	13.7(13.5, 13.9)	13.2(13.0, 13.4)	13.1(12.5, 13.7)
	551	632	763	2,060	2,042	299
UI	11.9(11.4, 12.4)	11.2(10.7, 11.6)	10.5(9.9, 11.1)	12.3(12.0, 12.5)	12.8(12.4, 13.1)	15.0(14.0, 15.9)
	530	622	679	1,888	1,660	284

Supplementary Table S13. Skill value with experience-awareness. This table lists the value of different skills with different lengths of experiences, formed as *mean (95% confidence interval)*. The sample size of each skill-experience pair is listed below the statistics.

Skill	Graduate	0-1	1-3	3-5	5-10	10+
Project Management	10.2(9.4, 10.9)<br>123	10.1(9.2, 11.0)<br>81	11.4(11.3, 11.6)<br>2,663	15.5(15.4, 15.6)<br>7,477	20.9(20.7, 21.1)<br>4,230	26(24.7, 27.3)<br>148
Architecture	16.4(15.9, 16.8)<br>872	17.0(16.1, 17.9)<br>269	19.9(19.8, 20.1)<br>11,774	25.7(25.6, 25.8)<br>31,584	34.0(33.9, 34.2)<br>16,020	38.3(37.1, 39.4)<br>429
Machine Learning	16.8(16.4, 17.2)<br>683	19.5(18.5, 20.5)<br>137	24.2(24.0, 24.4)<br>4,648	27.9(27.7, 28.0)<br>7,434	32.0(31.7, 32.4)<br>1,877	37.3(35.8, 38.8)<br>44
Golang	16.0(15.3, 16.7)<br>225	19.4(18.0, 20.8)<br>71	21.8(21.5, 22.1)<br>2,262	25.9(25.7, 26.1)<br>5,471	29.0(28.7, 29.3)<br>1,797	32.0(29.1, 34.0)<br>26
Algorithm	12.8(12.5, 13.1)<br>1,532	16.4(15.8, 17.1)<br>323	18.9(18.8, 19.1)<br>10,740	22.2(22.1, 22.3)<br>19,963	25.2(24.9, 25.4)<br>5,788	27.2(25.5, 29.0)<br>85
Hand Drawing	4.4(3.9, 5.0)<br>142	5.4(4.8, 6.0)<br>77	7.2(7.1, 7.4)<br>2,010	10.6(10.5, 10.8)<br>2,171	13.5(12.9, 14.0)<br>381	×
Photoshop	2.4(2.0, 2.7)<br>342	4.8(4.2, 5.3)<br>207	6.7(6.6, 6.9)<br>4,539	9.9(9.7, 10.1)<br>4,112	13.8(13.2, 14.4)<br>739	×
Vision Design	9.2(8.5, 9.9)<br>126	7.7(6.9, 8.4)<br>63	10.2(10.0, 10.4)<br>2,072	14.0(13.9, 14.2)<br>2,929	17.7(17.3, 18.1)<br>709	×
UI	5.9(5.1, 6.7)<br>98	5.6(4.5, 6.8)<br>46	9.7(9.4, 9.9)<br>1,803	13.3(13.0, 13.5)<br>2,711	17.7(17.0, 18.3)<br>621	×

Supplementary Table S14. Statistics for boxplots of company-aware IT skill value. This table lists the mean, lower quartile, median and upper quartile of the IT skill value for different companies.

Company	Skill	Sample Size	Mean	Lower Quartile	Median	Upper Quartile
Baidu	Algorithm	2,045	17.7	13.0	17.0	20.2
	Architecture	1,723	24.1	18.6	21.7	24.6
	Database	890	19.2	13.8	16.9	19.3
	Java	1,434	19.4	13.7	15.6	21.3
	Linux	1,803	18.0	12.5	14.8	20.9
	Python	1,708	19.2	15.3	16.8	19.8
ByteDance	Algorithm	1,262	28.7	26.1	28.5	30.1
	Architecture	1,087	30.9	25.4	28.4	32.0
	Database	493	17.5	13.4	16.3	17.1
	Java	858	21.0	15.8	18.9	20.7
	Linux	755	18.6	15.2	17.1	18.4
	Python	1,631	23.9	21.3	23.6	25.2
JD.com	Algorithm	577	23.4	19.2	21.4	24.4
	Architecture	782	30.5	22.2	27.6	34.0
	Database	443	16.1	9.8	13.0	19.3
	Java	813	24.8	16.8	20.4	29.8
	Linux	601	16.7	11.7	13.7	16.3
	Python	607	23.0	18.1	21.1	23.9
Meituan-Dianping	Algorithm	1,546	22.4	17.9	21.2	24.5
	Architecture	2,212	34.5	26.8	32.0	38.0
	Database	1,384	17.9	10.9	15.4	19.7
	Java	2,631	26.7	20.6	23.5	27.9
	Linux	1,191	18.5	12.3	17.2	22.9
	Python	1,466	24.0	19.8	23.5	25.2
Tencent	Algorithm	1,316	21.4	18.0	21.4	23.4
	Architecture	1,530	29.7	22.9	27.9	33.0
	Database	872	17.9	11.2	14.3	19.8
	Java	1,051	23.7	18.8	20.1	24.9
	Linux	1,784	15.3	11.2	13.3	16.1
	Python	1,250	17.6	12.3	17.2	19.3

Supplementary Table S15. Statistics for boxplots of company-aware designer skill value. This table lists the mean, lower quartile, median and upper quartile of the designer skill value for different companies.

Company	Skill	Sample Size	Mean	Lower Quartile	Median	Upper Quartile
Baidu	Hand Drawing	35	17.1	12.6	16.7	19.8
	Photoshop	66	14.4	10.3	12.9	16.3
	Vision Design	64	13.4	10.3	14.2	16.2
	illustrator	34	13.9	13.2	13.5	15.0
ByteDance	Hand Drawing	208	8.4	6.3	8.7	10.9
	Photoshop	370	11.4	7.8	13.1	15.4
	Vision Design	255	14.8	12.1	15.1	17.5
	illustrator	58	6.2	4.7	6.1	7.3
Meituan-Dianping	Hand Drawing	29	13.4	10.1	12.7	13.7
	Photoshop	28	9.6	7.3	10.1	12.2
	Vision Design	106	17.5	14.3	16.4	19.0
	illustrator	14	7.3	0.8	7.2	11.2
Tencent	Hand Drawing	36	13.9	11.3	13.2	14.4
	Photoshop	79	14.9	11.9	14.0	15.9
	Vision Design	75	15.7	12.5	16.3	16.4
	illustrator	21	9.5	5.9	10.2	14.2

Supplementary Table S16. Value of IT skills. Corresponding to the wordcloud that shows value of Machine Learning and Data Mining skills, this table lists the value statistics.

Skill	Value	Skill	Value	Skill	Value
Topic Model	38.4	Language Model	26.1	Cuda	19.7
Naive Bayes	37.0	Kalman Filter	25.8	Neural Network	19.2
DNN	36.4	Deep Belief Network	25.7	Intelligent Driving	18.9
Image Analysis	35.8	Deeplearning4j	25.5	Text Recognition	18.8
Graph Algorithm	35.2	Deep Learning	25.5	Multivariable Regression	18.4
Gradient Descent	33.2	CTR	25.3	Theano	18.3
Voiceprint Recognition	33.2	Image Retrieval	25.3	Decision Tree	18.2
Matrix Calculation	32.3	OCR	24.9	Beautifulsoup	17.6
NLTK	30.9	TensorFlow	24.7	User Behavior Analysis	16.7
Acoustic Modeling	30.4	Computational Linguistics	24.6	Logistic Regression	16.7
Machine Translation	30.4	LDA	24.3	Unsupervised Learning	16.6
Computational Advertising	30.2	Recommender System	24.2	Data Retrieval	16.4
Markov	30.2	Vision Algorithm	24.2	jieba	16.3
CRNN	30.1	Speech Recognition	24.2	Speech Technology	16.2
GAN	29.6	Reinforcement Learning	23.9	Linear Regression	15.8
xgboost	29.2	Public Opinion Analysis	23.8	Knowledge Discovery	15.8
POS Analysis	29.2	pyTorch	23.5	Genetic Algorithm	15.6
HCI	28.9	Knowledge Graph	23.5	Face Recognition	15.5
3D Vision	28.5	Information Retrieval	23.4	Image Fusion	15.4
PLSA	28.0	Machine Learning Library	23.1	Matrix Factorization	13.2
Graphical Model	28.0	Face Detection	21.9	Edge Detection	12.9
SVD	27.8	SVM	21.3	Game Theory	12.8
Kmeans	27.6	Seq2Seq	21.1	Voice Signal	12.1
Embedding	27.0	Text Mining	20.9	PageRank	11.8
LSTM	26.9	Text Classification	20.7	Gensim	11.8
Information Theory	26.8	Bayes	20.6	Multi-task	11.2
Image Segmentation	26.5	Probabilistic Graphical Model	20.2	Variance Analysis	11.1
Machine Learning	26.3	Image Matching	20.0	Mathematical Calculation	10.8
KNN	26.2	LibSvm	19.9	LSA	9.9

Supplementary Table S17. Domination of IT skills. Corresponding to the wordcloud that shows domination of Machine Learning and Data Mining skills, this table lists the domination statistics.

Skill	Dom (%)	Skill	Dom (%)	Skill	Dom (%)
Multivariable Regression	46.0	Gradient Descent	15.0	Theano	9.2
Unsupervised Learning	37.8	PLSA	14.5	Image Analysis	9.2
Image Fusion	34.8	Beautifulsoup	13.4	Logistic Regression	9.0
Mathematical Calculation	32.7	Probabilistic Graphical Model	13.0	Machine Translation	8.9
Data Retrieval	31.2	Text Recognition	12.9	Speech Recognition	8.7
Speech Technology	30.1	Intelligent Driving	12.7	Cuda	8.6
POS Analysis	29.5	Knowledge Discovery	11.8	Computational Advertising	8.5
Information Theory	28.7	Kmeans	11.7	OCR	8.1
Variance Analysis	27.0	Face Recognition	11.7	Naive Bayes	8.0
Image Matching	26.7	User Behavior Analysis	10.9	NLTK	7.9
Matrix Factorization	26.4	Machine Learning Library	10.9	xgboost	7.8
Voice Signal	25.4	Image Segmentation	10.5	Text Mining	7.8
Matrix Calculation	25.2	Image Retrieval	10.5	Recommender System	7.8
Markov	24.3	Face Detection	10.4	Vision Algorithm	7.6
Computational Linguistics	23.8	3D Vision	10.3	KNN	7.6
LSA	23.2	Kalman Filter	10.2	Graphical Model	7.6
Genetic Algorithm	21.3	Voiceprint Recognition	10.2	LSTM	7.5
PageRank	20.7	Deep Belief Network	10.1	TensorFlow	7.5
Edge Detection	20.6	Text Classification	9.8	Knowledge Graph	7.4
LibSvm	19.9	CTR	9.8	Reinforcement Learning	7.2
Multi-task	19.7	Neural Network	9.7	Deep Learning	7.1
Gensim	19.4	HCI	9.6	Information Retrieval	7.0
Topic Model	18.7	Public Opinion Analysis	9.5	SVD	7.0
Graph Algorithm	18.2	Decision Tree	9.5	LDA	6.8
Embedding	17.9	Language Model	9.4	Bayes	6.7
Seq2Seq	17.7	Linear Regression	9.4	Machine Learning	6.6
jieba	16.6	GAN	9.3	pyTorch	6.5
Game Theory	15.9	CRNN	9.3	DNN	6.5
Deeplearning4j	15.8	Acoustic Modeling	9.2	SVM	5.8

Supplementary Table S18. Contribution of IT skills. Corresponding to the wordcloud that shows contribution of Machine Learning and Data Mining skills, this table lists the contribution statistics.

Skill	Contribution	Skill	Contribution	Skill	Contribution
POS Analysis	8.508	Naive Bayes	2.853	DNN	2.022
Topic Model	7.906	Kmeans	2.803	Text Recognition	1.992
Markov	7.811	Image Segmentation	2.778	Public Opinion Analysis	1.963
Information Theory	7.455	Image Retrieval	2.747	SVD	1.959
Matrix Calculation	7.375	Machine Translation	2.737	Vision Algorithm	1.923
Multivariable Regression	7.208	3D Vision	2.685	LSTM	1.922
Unsupervised Learning	6.420	Probabilistic Graphical Model	2.638	Recommender System	1.902
Computational Linguistics	5.901	LSA	2.601	KNN	1.900
Image Matching	5.664	CRNN	2.568	Text Classification	1.877
Image Fusion	5.493	Computational Advertising	2.521	Deep Belief Network	1.868
Graph Algorithm	5.397	Beautifulsoup	2.506	Deep Learning	1.853
Speech Technology	4.999	CTR	2.487	Neural Network	1.809
Data Retrieval	4.992	Edge Detection	2.481	TensorFlow	1.787
Gradient Descent	4.921	Game Theory	2.453	User Behavior Analysis	1.768
Embedding	4.894	HCI	2.444	Face Recognition	1.764
PLSA	4.054	Knowledge Discovery	2.397	Machine Learning	1.739
LibSvm	3.836	NLTK	2.342	Reinforcement Learning	1.718
Mathematical Calculation	3.740	Language Model	2.339	Information Retrieval	1.713
Voice Signal	3.694	Intelligent Driving	2.326	Theano	1.701
Matrix Factorization	3.489	Multi-task	2.323	LDA	1.691
Genetic Algorithm	3.365	xgboost	2.251	Knowledge Graph	1.686
Deeplearning4j	3.313	Machine Learning Library	2.251	pyTorch	1.653
Variance Analysis	3.289	Gensim	2.227	Linear Regression	1.600
Voiceprint Recognition	3.274	Kalman Filter	2.217	Text Mining	1.598
jieba	2.995	Face Detection	2.159	Decision Tree	1.592
Seq2Seq	2.991	OCR	2.127	Logistic Regression	1.556
Image Analysis	2.977	Speech Recognition	2.085	Cuda	1.551
GAN	2.958	PageRank	2.082	Bayes	1.510
Acoustic Modeling	2.888	Graphical Model	2.081	SVM	1.121

Supplementary Table S19. Value of designer skills. Corresponding to the wordcloud that shows the value of the designer skills, this table lists the value statistics.

Skill	Value	Skill	Value	Skill	Value
Database	20.0	Spine	12.4	Art Design	8.3
UED	19.5	Front End Development	12.2	Web Side	8.2
UX	19.4	UI	12.2	Composition	8.2
Realistic Style	17.7	Mobile Game	11.8	Premiere	8.2
JavaScript	17.4	Aesthetic Basis	11.7	Design Software	8.2
Document Management	17.4	C4D	11.7	Android	8.2
Java	16.9	w3c	11.6	CSS	8.0
Product Planning	15.5	XHtml	11.5	PhotoShop	8.0
Interaction Eesign	15.4	Product Development	11.5	Human Body Structure	7.8
Web2.0	15.1	English	11.5	Mac	7.7
3D Modeling	15.1	Post Production	11.2	AI	7.7
PHP	15.0	Axure	11.1	Web Standards	7.5
Machine Design	14.9	GUI	11.1	Layout	7.5
IOS	14.8	Design Execution	11.1	JQuery	7.4
User Interaction	14.5	Interactive Prototypes	11.0	Indesign	7.3
Interface Interaction	14.4	AE	10.6	Overall Website Planning	7.3
Data Analysis	14.4	Operate	10.6	Office Software	7.3
product Design	14.1	Illustration	10.5	Logo	7.2
Game Art	14.1	Maya	10.4	Page Design	7.1
Image Design	14.1	Interface Design	10.3	Interior Decoration	7.1
Mobile UI	14.0	Design Requirement Analysis	10.3	Graphic Artist Designer	7.0
Brand Design	14.0	Painting	10.2	iphone	7.0
Design Theory	13.7	Mobile Product Design	10.2	Clip	6.9
Software Development	13.6	ICON	10.2	Flash	6.9
Design Code	13.6	Business Design	10.0	CorelDraw	6.8
Dynamic Effect	13.5	Original Painting	9.9	H5	6.8
Project Management	13.5	Brand Image	9.7	PPT	6.8
Game Interface	13.3	Game Development Process	9.7	Visual Marketing	6.6
Unity	13.3	Advertising Creativity	9.5	Interior Design	6.5
Ajax	13.3	Creative Design	9.5	customer Service	6.5
Sketch	13.1	DIV	9.5	Product Packaging	6.2
MySql	13.1	Online Retailers	9.5	Webpage Making	6.2
User Research	13.1	Modeling Ability	9.4	Drafting	6.1
Unity3d	13.0	Animation	9.3	Website Design	6.0
Internet Product Design	12.9	Font Design	9.2	Image Processing	5.7
Visual Design	12.9	3Dmax	9.2	Print Advertisement	5.7
APP	12.9	Construction Plans	9.0	Revision of Drawings	5.4
Human-Computer Interaction	12.8	Construction Technology	9.0	Art Software	5.0
Computer	12.8	Printing Process	8.9	CDR	4.8
UE	12.7	Hand Painted	8.9	Website Construction	4.4
UCD	12.6	Mapping	8.7	Dreamweaver	4.3
Product Interaction	12.5	Illustrator	8.6	HTML	4.1
Visio	12.4	CAD	8.5	Cutaway	3.6

Supplementary Table S20. Domination of designer skills. Corresponding to the wordcloud that shows the domination of designer skills, this table lists the domination statistics.

Skill	Dom(%)	Skill	Dom(%)	Skill	Dom(%)
Machine Design	21.3	Animation	13.2	User Research	11.8
MySql	21.0	CDR	13.2	Page Design	11.8
Software Development	19.0	PhotoShop	13.1	Web Side	11.8
Database	18.7	Game Interface	13.1	AE	11.8
Original Painting	17.9	Operate	13.0	Visual Design	11.7
Java	17.5	Graphic Artist Designer	13.0	ICON	11.7
Spine	16.4	Design Requirement Analysis	13.0	Design Theory	11.7
Product Development	16.4	H5	12.9	Product Interaction	11.6
3Dmax	16.3	Indesign	12.9	Flash	11.6
Unity3d	16.3	Interior Design	12.9	Post Production	11.6
Maya	16.3	CorelDraw	12.8	Web Standards	11.6
Unity	16.3	Font Design	12.8	Art Software	11.6
CAD	16.2	Composition	12.7	APP	11.5
Realistic Style	16.1	Brand Image	12.7	Interface Interaction	11.5
Sketch	16.1	UI	12.7	Business Design	11.5
Mapping	16.0	Illustration	12.6	Illustrator	11.4
Interactive Prototypes	15.6	Creative Design	12.6	Interface Design	11.4
Construction Technology	15.6	Printing Process	12.5	Product Planning	11.4
Modeling Ability	15.4	PPT	12.5	Dynamic Effect	11.2
Painting	15.0	AI	12.5	Axure	11.2
3D Modeling	14.8	Product Packaging	12.5	Design Code	11.2
Human Body Structure	14.8	Advertising Creativity	12.4	Dreamweaver	11.0
Office Software	14.8	Mac	12.4	UE	11.0
Data Analysis	14.7	English	12.4	Image Design	10.8
Game Art	14.7	Print Advertisement	12.3	UED	10.7
Clip	14.7	GUI	12.3	Website Design	10.7
Drafting	14.6	HTML	12.3	Overall Website Planning	10.7
Interior Decoration	14.5	Mobile Product Design	12.2	Cutaway	10.5
Game Development Process	14.1	Ajax	12.2	Webpage Making	10.5
Design Execution	14.1	Interaction Eesign	12.2	Visual Marketing	10.2
Revision of Drawings	14.1	Website Construction	12.2	Front End Development	10.2
UCD	14.1	Computer	12.2	Android	10.1
Mobile Game	14.0	Product Design	12.1	Internet Product Design	10.1
C4D	13.9	Document Management	12.1	User Interaction	10.1
iphone	13.9	Visio	12.1	CSS	9.6
Art Design	13.7	Design Software	12.1	IOS	9.4
Image Processing	13.7	customer Service	12.1	w3c	9.2
Premiere	13.7	Logo	12.0	XHtml	9.1
Hand Painted	13.4	Layout	12.0	JQuery	9.1
Aesthetic Basis	13.3	Human-Computer Interaction	12.0	Mobile UI	8.9
Brand Design	13.2	Project Management	11.9	JavaScript	8.3
Online Retailers	13.2	PHP	11.9	DIV	8.1
Construction Plans	13.2	UX	11.9	Web2.0	6.7

Supplementary Table S21. Contribution of designer skills. Corresponding to the wordcloud that shows the contribution of designer skills, this table lists the contribution statistics.

Skill	Contribution	Skill	Contribution	Skill	Contribution
Database	3.93	Visual Design	1.49	Composition	1.01
Machine Design	3.20	APP	1.48	Clip	0.98
Java	3.16	Sketch	1.45	PhotoShop	0.98
Realistic Style	2.74	Design Execution	1.45	Web Side	0.96
Software Development	2.65	Image Design	1.42	Design Software	0.96
MySql	2.56	User Interaction	1.42	AI	0.95
UX	2.28	JavaScript	1.40	Illustrator	0.94
Data Analysis	2.18	Modeling Ability	1.40	Interior Decoration	0.94
Unity3d	2.16	English	1.40	XHtml	0.94
Unity	2.15	IOS	1.40	Web2.0	0.93
Document Management	2.13	UI	1.40	Indesign	0.93
3D Modeling	2.11	Game Development Process	1.39	Mac	0.92
Game Art	2.09	Mapping	1.37	customer Service	0.91
UED	2.09	GUI	1.36	Layout	0.88
Spine	2.06	Aesthetic Basis	1.36	H5	0.88
Product Development	1.95	Operate	1.35	Graphic Artist Designer	0.87
Interaction Eesign	1.87	Construction Technology	1.35	Drafting	0.86
Brand Design	1.86	CAD	1.34	Logo	0.86
Interactive Prototypes	1.83	Illustration	1.34	CorelDraw	0.84
PHP	1.82	UE	1.33	Product Packaging	0.84
UCD	1.81	Design Requirement Analysis	1.33	Page Design	0.83
Original Painting	1.78	Construction Plans	1.32	PPT	0.83
Product Planning	1.75	Mobile Product Design	1.28	Android	0.82
Product Design	1.73	Internet Product Design	1.28	Image Processing	0.79
Game Interface	1.71	Axure	1.26	Web Standards	0.78
Maya	1.67	Online Retailers	1.24	Flash	0.78
Mobile Game	1.65	Post Production	1.24	DIV	0.78
Ajax	1.65	AE	1.24	CSS	0.77
Design Theory	1.64	Human Body Structure	1.23	iphone	0.75
C4D	1.63	Brand Image	1.21	Revision of Drawings	0.73
Project Management	1.61	Animation	1.20	Interior Design	0.72
Human-Computer Interaction	1.59	Creative Design	1.19	Visual Marketing	0.72
Dynamic Effect	1.55	w3c	1.18	Print Advertisement	0.68
Computer	1.55	Hand Painted	1.17	Website Design	0.64
Painting	1.54	Advertising Creativity	1.16	Webpage Making	0.62
User Research	1.53	ICON	1.16	JQuery	0.61
Interface Interaction	1.53	Art Design	1.15	CDR	0.61
Design Code	1.52	Interface Design	1.15	Overall Website Planning	0.56
Business Design	1.51	Front End Development	1.14	Art Software	0.55
Visio	1.51	Font Design	1.10	Website Construction	0.53
Mobile UI	1.51	Printing Process	1.08	HTML	0.49
3Dmax	1.50	Premiere	1.05	Dreamweaver	0.44
Product Interaction	1.50	Office Software	1.02	Cutaway	0.39

Supplementary Table S22. **Statistics in boxplots of salary distribution.** This table lists the average, lower quartile, median and upper quartile of the salary distribution in the IT dataset and the designer dataset.

Dataset	Time	Bound	Sample Size	Mean	Lower Quartile	Median	Upper Quartile
IT	2016-H2	Lower	84,233	10.76	7	10	15
		Upper	83,448	18.42	11	16	25
	2017-H1	Lower	85,078	11.20	7	10	15
		Upper	84,762	19.21	12	18	25
	2017-H2	Lower	93,899	11.84	8	10	15
		Upper	93,625	20.42	12	20	30
	2018-H1	Lower	254,998	11.63	7	10	15
		Upper	254,221	19.97	12	18	25
	2018-H2	Lower	242,965	12.91	8	10	15
		Upper	242,730	22.12	13	20	30
	2019-H1	Lower	36,758	13.83	8	12	18
		Upper	36,724	23.78	15	20	30
Designer	2016-H2	Lower	10,875	7.328	5	6	10
		Upper	10,875	12.46	8	10	15
	2017-H1	Lower	10,280	7.20	4	6	10
		Upper	10,780	12.17	8	10	15
	2017-H2	Lower	10,504	7.43	4	6	10
		Upper	10,259	12.56	8	10	16
	2018-H1	Lower	33,558	7.35	4	6	10
		Upper	10,484	12.39	8	10	15
	2018-H2	Lower	29,342	8.095	5	7	10
		Upper	33,466	13.60	8	12	18
	2019-H1	Lower	3,785	8.67	5	8	10
		Upper	3,784	14.52	8	12	20

Supplementary Table S23. Statistics in boxplots of value distribution under different job positions. This table lists the mean, lower quartile, median and upper quartile of 4 skills' value distribution in 6 job positions.

Skill	Job	Sample Size	Mean	Lower Quartile	Median	Upper Quartile
Database	Android Engineer	364	12.30	8.47	10.77	13.89
	Crawler Engineer	99	12.88	8.43	10.73	13.75
	Data Analyzer	448	10.55	8.04	10.07	12.56
	Data Engineer	803	14.41	8.67	11.84	16.89
	PHP Engineer	1,623	13.15	8.84	11.67	15.21
	Test Engineer	1,372	11.65	7.95	10.39	13.44
Java	Android Engineer	2,000	15.58	11.53	14.65	18.19
	Crawler Engineer	141	19.18	11.84	16.30	22.64
	Data Analyzer	139	15.40	10.90	13.91	17.65
	Data Engineer	1,589	18.85	12.59	16.06	21.58
	PHP Engineer	296	20.80	14.00	18.08	25.29
	Test Engineer	1,734	15.19	10.87	13.73	17.69
Linux	Android Engineer	154	12.73	8.80	11.13	14.19
	Crawler Engineer	105	13.51	10.02	11.34	14.51
	Data Analyzer	126	11.79	8.57	11.31	14.45
	Data Engineer	878	14.51	9.49	12.35	16.27
	PHP Engineer	1,370	12.40	8.83	11.41	14.26
	Test Engineer	1,442	11.51	8.40	10.72	13.51
Python	Android Engineer	38	18.41	13.02	16.31	19.59
	Crawler Engineer	175	16.32	12.58	16.10	18.53
	Data Analyzer	968	16.51	13.26	16.41	19.65
	Data Engineer	1,347	17.96	13.70	16.97	20.52
	PHP Engineer	400	17.11	13.95	16.95	20.01
	Test Engineer	1,545	16.13	12.25	15.87	19.08

Supplementary References

1. Murphy, K. M. & Welch, F. Occupational change and the demand for skill, 1940-1990. *The American Economic Review* **83**, 122–126 (1993).
2. Maurin, E. & Thesmar, D. Changes in the functional structure of firms and the demand for skill. *Journal of labor economics* **22**, 639–664 (2004).
3. Beaudry, P., Green, D. A. & Sand, B. M. The great reversal in the demand for skill and cognitive tasks. *Journal of Labor Economics* **34**, S199–S247 (2016).
4. Kankaraš, M., Montt, G., Paccagnella, M., Quintini, G. & Thorn, W. Skills matter: Further results from the survey of adult skills. oecd skills studies. *OECD Publishing* (2016).
5. Desjardins, R. *et al.* Oecd skills outlook 2013: First results from the survey of adult skills. *Journal of Applied Econometrics* **30**, 1144–1168 (2013).
6. Arnett, K. P. & Litecky, C. R. Career path development for the most wanted skills in the mis job market. *Journal of Systems Management* (1994).
7. Yan, R. *et al.* Interview choice reveals your preference on the market: To improve job-resume matching through profiling memories. In *ACM KDD 2019* (2019).
8. Zhu, C. *et al.* Person-job fit: Adapting the right talent for the right job with joint representation learning. *ACM Transactions on Management Information Systems (TMIS)* (2018).
9. Boselli, R., Cesarini, M., Mercorio, F. & Mezzanzanica, M. Using machine learning for labour market intelligence. In *Joint European Conference on Machine Learning and Knowledge Discovery in Databases*, 330–342 (Springer, 2017).
10. for the Development of Vocational Training (Cedefop), E. C. The online job vacancy market in the eu: driving forces and emerging trends (2019).
11. for the Development of Vocational Training (Cedefop), E. C. Online job vacancies and skills analysis: a cedefop pan-european approach (2019).
12. Boselli, R. *et al.* Wolmis: a labor market intelligence system for classifying web job vacancies. *Journal of intelligent information systems* **51**, 477–502 (2018).
13. Lovaglio, P. G., Cesarini, M., Mercorio, F. & Mezzanzanica, M. Skills in demand for ict and statistical occupations: Evidence from web-based job vacancies. *Statistical Analysis and Data Mining: The ASA Data Science Journal* **11**, 78–91 (2018).
14. Colombo, E., Mercorio, F. & Mezzanzanica, M. Applying machine learning tools on web vacancies for labour market and skill analysis. *Terminator or the Jetsons? The Economics and Policy Implications of Artificial Intelligence* (2018).
15. Wu, X. *et al.* Trend-aware tensor factorization for job skill demand analysis. In *IJCAI 2019* (2019).
16. Marrara, S. *et al.* A language modelling approach for discovering novel labour market occupations from the web. In *Proceedings of the International Conference on Web Intelligence*, 1026–1034 (2017).
17. Zhu, C., Zhu, H., Xiong, H., Ding, P. & Xie, F. Recruitment market trend analysis with sequential latent variable models. In *Proceedings of the 22nd ACM SIGKDD international conference on knowledge discovery and data mining*, 383–392 (2016).
18. Xu, T., Zhu, H., Zhu, C., Li, P. & Xiong, H. Measuring the popularity of job skills in recruitment market: A multi-criteria approach. In *AAAI 2018* (2018).
19. Dix-Carneiro, R. & Kovak, B. K. Trade liberalization and the skill premium: A local labor markets approach. *American Economic Review* **105**, 551–57 (2015).
20. Burstein, A. & Vogel, J. International trade, technology, and the skill premium. *Journal of Political Economy* **125**, 1356–1412 (2017).
21. Topel, R. H. Regional labor markets and the determinants of wage inequality. *The American Economic Review* **84**, 17–22 (1994).
22. Lazar, A. Income prediction via support vector machine. In *ICMLA*, 143–149 (Citeseer, 2004).
23. Blankmeyer, E., LeSage, J. P., Stutzman, J., Knox, K. J. & Pace, R. K. Peer-group dependence in salary benchmarking: a statistical model. *Managerial and Decision Economics* **32**, 91–104 (2011).

24. Lin, H. *et al.* Collaborative company profiling: Insights from an employee's perspective. *arXiv preprint arXiv:1712.02987* (2017).

25. Meng, Q., Zhu, H., Xiao, K. & Xiong, H. Intelligent salary benchmarking for talent recruitment: A holistic matrix factorization approach. In *IEEE ICDM 2018* (2018).

26. Mnih, V., Heess, N., Graves, A. *et al.* Recurrent models of visual attention. In *Advances in neural information processing systems*, 2204–2212 (2014).

27. Wang, F. *et al.* Residual attention network for image classification. In *Proceedings of the IEEE conference on computer vision and pattern recognition*, 3156–3164 (2017).

28. Bahdanau, D., Cho, K. & Bengio, Y. Neural machine translation by jointly learning to align and translate. *arXiv preprint arXiv:1409.0473* (2014).

29. Vaswani, A. *et al.* Attention is all you need. In *Advances in neural information processing systems*, 5998–6008 (2017).

30. Yang, Z. *et al.* Hierarchical attention networks for document classification. In *Proceedings of the 2016 conference of the North American chapter of the association for computational linguistics: human language technologies*, 1480–1489 (2016).

31. Lu, P. *et al.* R-vqa: learning visual relation facts with semantic attention for visual question answering. In *Proceedings of the 24th ACM SIGKDD International Conference on Knowledge Discovery & Data Mining*, 1880–1889 (2018).

32. Fan, C. *et al.* Multi-horizon time series forecasting with temporal attention learning. In *Proceedings of the 25th ACM SIGKDD International Conference on Knowledge Discovery & Data Mining*, 2527–2535 (2019).

33. Lin, H., Bai, R., Jia, W., Yang, X. & You, Y. Preserving dynamic attention for long-term spatial-temporal prediction. In *Proceedings of the 26th ACM SIGKDD International Conference on Knowledge Discovery & Data Mining*, 36–46 (2020).

34. Petar, V. *et al.* Graph attention networks. In *International Conference on Learning Representations* (2018).

35. Wang, X., He, X., Cao, Y., Liu, M. & Chua, T.-S. Kgat: Knowledge graph attention network for recommendation. In *Proceedings of the 25th ACM SIGKDD International Conference on Knowledge Discovery & Data Mining*, 950–958 (2019).

36. Choi, E., Bahadori, M. T., Song, L., Stewart, W. F. & Sun, J. Gram: graph-based attention model for healthcare representation learning. In *Proceedings of the 23rd ACM SIGKDD International Conference on Knowledge Discovery and Data Mining*, 787–795 (2017).

37. Wang, X. *et al.* Heterogeneous graph attention network. In *The World Wide Web Conference*, 2022–2032 (2019).

38. Luo, J., Ye, M., Xiao, C. & Ma, F. Hitanet: Hierarchical time-aware attention networks for risk prediction on electronic health records. In *Proceedings of the 26th ACM SIGKDD International Conference on Knowledge Discovery & Data Mining*, 647–656 (2020).

39. Zhang, Y., Li, Y., Zhou, X., Kong, X. & Luo, J. Curb-gan: Conditional urban traffic estimation through spatio-temporal generative adversarial networks. In *Proceedings of the 26th ACM SIGKDD International Conference on Knowledge Discovery & Data Mining*, 842–852 (2020).

40. Ghosh, A., Heffernan, N. & Lan, A. S. Context-aware attentive knowledge tracing. In *Proceedings of the 26th ACM SIGKDD International Conference on Knowledge Discovery & Data Mining*, 2330–2339 (2020).

41. Kim, S. *et al.* Date: Dual attentive tree-aware embedding for customs fraud detection. In *Proceedings of the 26th ACM SIGKDD International Conference on Knowledge Discovery & Data Mining*, 2880–2890 (2020).

42. Oelberger, C. R., Fechter, A.-M. & McWha-Hermann, I. Managing human resources in international ngos. *The Nonprofit Human Resource Management Handbook: From Theory to Practice* 285–303 (2017).

43. Medoff, J. L. & Abraham, K. G. Are those paid more really more productive? the case of experience. *Journal of Human resources* 186–216 (1981).

44. Rosenberg, N. On technological expectations. *The Economic Journal* **86**, 523–535 (1976).

45. Bessen, J. Employers aren't just whining–the 'skills gap'is real. *Harvard Business Review* **25** (2014).

46. Davenport, T. H., Harris, J. & Shapiro, J. Competing on talent analytics. *Harvard business review* **88**, 52–58 (2010).

47. Ozcaglar, C. *et al.* Entity personalized talent search models with tree interaction features. In *The World Wide Web Conference*, 3116–3122 (2019).

48. Geyik, S. C., Ambler, S. & Kenthapadi, K. Fairness-aware ranking in search & recommendation systems with application to linkedin talent search. In *Proceedings of the 25th ACM SIGKDD International Conference on Knowledge Discovery & Data Mining*, 2221–2231 (2019).

49. Qin, C. *et al.* Duerquiz: A personalized question recommender system for intelligent job interview. In *ACM KDD 2019* (2019).

50. Javed, F., Hoang, P., Mahoney, T. & McNair, M. Large-scale occupational skills normalization for online recruitment. In *Twenty-ninth IAAI conference* (2017).

51. Sun, J. Jieba chinese word segmentation tool. *Accessed: Jun* **25**, 2018 (2012).

52. Radim Rehurek and Petr Sojka. Software Framework for Topic Modelling with Large Corpora. In *Proceedings of the LREC 2010 Workshop on New Challenges for NLP Frameworks*, 45–50 (ELRA, Valletta, Malta, 2010).

53. Bar-Yehuda, R. & Even, S. A linear-time approximation algorithm for the weighted vertex cover problem. *Journal of Algorithms* **2**, 198–203 (1981).
