# Supplementary material for: Market-oriented job skill valuation with cooperative composition neural network
Source: Nat Commun. 2021 Mar 31;12:1992. doi: 10.1038/s41467-021-22215-y (PMC8012576; doi:10.1038/s41467-021-22215-y)
Supplement: Supplementary file 2 — Reporting Summary [file 41467_2021_22215_MOESM2_ESM.pdf]

nature research

Corresponding author(s): Fuzhen Zhuang
Fengshu Zhu
Hui Xiong

Last updated by author(s): Feb 25, 2021

Reporting Summary

Nature Research wishes to improve the reproducibility of the work that we publish. This form provides structure for consistency and transparency in reporting. For further information on Nature Research policies, see our Editorial Policies and the Editorial Policy Checklist.

Statistics

For all statistical analyses, confirm that the following items are present in the figure legend, table legend, main text, or Methods section.

n/a	Confirmed	
☐	☒	The exact sample size (n) for each experimental group/condition, given as a discrete number and unit of measurement
☐	☒	A statement on whether measurements were taken from distinct samples or whether the same sample was measured repeatedly
☐	☒	The statistical test(s) used AND whether they are one- or two-sided<br>*Only common tests should be described solely by name; describe more complex techniques in the Methods section.*
☒	☐	A description of all covariates tested
☐	☒	A description of any assumptions or corrections, such as tests of normality and adjustment for multiple comparisons
☐	☒	A full description of the statistical parameters including central tendency (e.g. means) or other basic estimates (e.g. regression coefficient) AND variation (e.g. standard deviation) or associated estimates of uncertainty (e.g. confidence intervals)
☐	☒	For null hypothesis testing, the test statistic (e.g. F, t, r) with confidence intervals, effect sizes, degrees of freedom and P value noted<br>*Give P values as exact values whenever suitable.*
☒	☐	For Bayesian analysis, information on the choice of priors and Markov chain Monte Carlo settings
☒	☐	For hierarchical and complex designs, identification of the appropriate level for tests and full reporting of outcomes
☒	☐	Estimates of effect sizes (e.g. Cohen's d, Pearson's r), indicating how they were calculated

Our web collection on statistics for biologists contains articles on many of the points above.

Software and code

Policy information about availability of computer code

Data collection	The dataset was provided by our industrial partner.
Data analysis	We used numpy, matplotlib, seaborn and scikit-learn package in Python for data analysis.

For manuscripts utilizing custom algorithms or software that are central to the research but not yet described in published literature, software must be made available to editors and reviewers. We strongly encourage code deposition in a community repository (e.g. GitHub). See the Nature Research guidelines for submitting code & software for further information.

Data

Policy information about availability of data

All manuscripts must include a data availability statement. This statement should provide the following information, where applicable:

- Accession codes, unique identifiers, or web links for publicly available datasets
- A list of figures that have associated raw data
- A description of any restrictions on data availability

The job posting data that support the findings of this studyare available in figshare with the identifier "10.6084/m9.figshare.14060498". Source data for all the figures and tables in this paper are provided with this paper. All data generated or analysed during this study are included in this published article (and its supplementary information files).

Field-specific reporting

Please select the one below that is the best fit for your research. If you are not sure, read the appropriate sections before making your selection.

☐ Life sciences ☒ Behavioural & social sciences ☐ Ecological, evolutionary & environmental sciences

For a reference copy of the document with all sections, see nature.com/documents/nr-reporting-summary-flat.pdf

Behavioural & social sciences study design

All studies must disclose on these points even when the disclosure is negative.

Study description	Quantitative
Research sample	The research samples are online job advertisements collected from the Internet.
Sampling strategy	We did not use sampling strategies and used all the data we have collected for analysis, after filtering out the outliers. The detailed information can be found in Method section.
Data collection	The dataset was provided by our industrial partner.
Timing	Our data range from July 2016 to June 2019.
Data exclusions	We have filtered out the job postings whose upper-bound or lower-bound salary are outliers in the dataset. The rationale is that the online job postings contain some noise, when some recruiters may fill in fake salary that is abnormally high to attract job seekers' attention. Meanwhile, we filtered out small companies and cities that rarely publish job postings on the website to further reduce data noise. The detailed information can be found in the Method section.
Non-participation	Not applicable, since our data are job postings collected from the Internet.
Randomization	Not applicable, since our data are job postings collected from the Internet.

Reporting for specific materials, systems and methods

We require information from authors about some types of materials, experimental systems and methods used in many studies. Here, indicate whether each material, system or method listed is relevant to your study. If you are not sure if a list item applies to your research, read the appropriate section before selecting a response.

Materials & experimental systems

n/a	Involved in the study	
☒	☐	Antibodies
☒	☐	Eukaryotic cell lines
☒	☐	Palaeontology and archaeology
☒	☐	Animals and other organisms
☒	☐	Human research participants
☒	☐	Clinical data
☒	☐	Dual use research of concern

Methods

n/a	Involved in the study	
☒	☐	ChIP-seq
☒	☐	Flow cytometry
☒	☐	MRI-based neuroimaging
